# Tumor Derived Extracellular Vesicles Drive T Cell Exhaustion in Tumor Microenvironment through Sphingosine Mediated Signaling and Impacting Immunotherapy Outcomes in Ovarian Cancer

**DOI:** 10.1002/advs.202104452

**Published:** 2022-03-15

**Authors:** Prachi Gupta, Ishaque Pulikkal Kadamberi, Sonam Mittal, Shirng‐Wern Tsaih, Jasmine George, Sudhir Kumar, Dileep K. Vijayan, Anjali Geethadevi, Deepak Parashar, Paytsar Topchyan, Lindsey McAlarnen, Brian F Volkman, Weiguo Cui, Kam Y. J. Zhang, Dolores Di Vizio, Pradeep Chaluvally‐Raghavan, Sunila Pradeep

**Affiliations:** ^1^ Department of Obstetrics and Gynecology Medical College of Wisconsin Milwaukee Wisconsin 53226 USA; ^2^ Laboratory for computational and structural biology Jubilee Center for Medical Research Thrissur Kerala 680006 India; ^3^ Laboratory for Structural Bioinformatics Center for Biosystems Dynamics Research Riken 230‐0045 Japan; ^4^ Department of Microbiology and Immunology MCW and Versiti Blood Research Institute Milwaukee Wisconsin 53226 USA; ^5^ Department of Biochemistry Medical College of Wisconsin Milwaukee 53226 USA; ^6^ Department of Surgery Pathology and Laboratory Medicine Samuel Oschin Comprehensive Cancer Institute Cedars‐Sinai Medical Center Los Angeles CA 90048 USA; ^7^ Department of Physiology Medical College of Wisconsin Milwaukee Wisconsin 53226 USA; ^8^ Medical College of Wisconsin Cancer Center Medical College of Wisconsin Milwaukee Wisconsin 53226 USA

**Keywords:** extracellular vesicles, immunotherapy, ovarian cancer, S1P, SPHK1, T cell exhaustion

## Abstract

SPHK1 (sphingosine kinase‐1) catalyzes the phosphorylation of sphingosine to sphingosine‐1‐phosphate (S1P), is found to be highly expressed in solid tumors. Here, extracellular vesicles (EVs) are identified as the key transporters of SPHK1 to the tumor microenvironment. Consequently, SPHK1‐packaged EVs elevate S1P levels in the tumor microenvironment, where S1P appears as an immunosuppressive agent. However, the exact mechanism of how S1P mediates its immunosuppressive effects in cancer is not understood. It is investigated that S1P can induce T cell exhaustion. S1P can also upregulate programmed death ligand‐1 (PDL‐1) expression through E2F1‐mediated transcription. Notably, an SPHK1 inhibitor PF543 improves T cell‐mediated cytotoxicity. Furthermore, combining PF543 with an anti‐PD‐1 antibody reduces tumor burden and metastasis more effectively than PF543 alone in vivo. These data demonstrate a previously unrecognized mechanism of how SPHK1‐packaged EVs contribute to the progression of ovarian cancer and thus present the potential clinical application of inhibiting SPHK1/S1P signaling to improve immune checkpoint blockage (anti‐PD‐1 antibody) therapy in ovarian cancer.

## Graphical abstract

The mechanism of immunosuppression through extracellular vesicles (EVs) in ovarian cancer is identified. Sphingosine kinase‐1 (SPHK1) secreted through EVs facilitates sphingosine‐1‐phosphate (S1P) synthesis in tumor microenvironment (TME) and elevates S1P. High S1P results in immune suppression through T cell‐exhaustion by upregulating programmed death ligand‐1 (PD‐L1) expression. SPHK1 inhibition improves the efficacy of immune checkpoint blockage therapy in ovarian cancer.

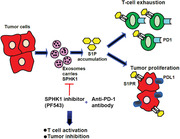

## 1 Introduction

Checkpoint immunotherapy trials such as programmed cell death protein‐1/programmed death ligand‐1 (PD‐1/PD‐L1) inhibitor therapy seemed promising for ovarian cancer patients at the beginning of their treatments. However, extensive studies showed fairly low response rates: <5% regardless of which agent was used to target PD‐1/PD‐L1.^[^ ^1^ ^]^ More recently, targeting immunometabolism as a potential mechanism for enhancing anti‐PD‐1 therapy was found to be effective against melanoma.^[^ ^2^ ^]^ One such mechanism identified to treat melanoma was an immune‐metabolite Sphingosine‐1‐phosphate (S1P).^[^ ^2^ ^]^

S1P is synthesized when sphingosine kinase‐1 (SPHK1) phosphorylates sphingosine. SPHK1/S1P signaling has been linked to cancer progression and metastasis,^[^ ^3^ ^]^ as it modulates many key oncogenic events, such as proliferation, migration, invasion, adhesion, angiogenesis, and apoptosis.^[^ ^4^ ^]^ Consistent with these observations, we found elevated S1P levels in high‐grade serous ovarian cancer patients’ plasma and high SPHK1 enzyme expression in tissue samples. Moreover, S1P signaling is also known to control immune cell trafficking.^[^ ^5^ ^]^ Recently, S1P was found to regulate upstream signaling that affects the metabolic fitness of T cells in the tumor microenvironment (TME).^[^ ^2^ ^]^ It was also shown that targeting S1P signaling improves tumor control in a dual fashion: by negatively controlling tumor growth and by increasing the antitumor activity of T cells.^[^ ^6^ ^]^

While it is well known that SPHK1 phosphorylates sphingosine intracellularly to form S1P,^[^ ^7^ ^]^ we recently made the surprising discovery that ovarian cancer cells release extracellular vesicles (EVs) carrying SPHK1 into the TME, allowing S1P to be generated outside the cancer cell. EVs are known to affect tumor progression by promoting metastasis, immune modulation, angiogenesis, and tissue regeneration.^[^ ^8^ ^]^ However, how EVs containing SPHK1 and therefore S1P elevation modulate the tumor microenvironment is unknown. In addition, there is a paucity of data on the effect of S1P on immune cell responsiveness to cancer cells.

While evaluating the mechanisms regulating S1P levels in the tumor microenvironment, we discovered that S1P upregulates PD‐L1 expression in tumor cells, activating T cell death mechanisms that exhaust cytotoxic T cell activity. These findings led us to combine PF543 (an SPHK1 inhibitor) with anti‐PD‐1 therapy in a syngeneic model of ovarian cancer. We found a significant reduction in tumor burden and improved survival in vivo. Using both in vivo and in vitro methods, we demonstrated that i) EVs‐SPHK1 (SPHK1‐packaged EVs) induce immune suppression in ovarian cancer and ii) targeting SPHK1 with a specific inhibitor, PF543, in combination with anti‐PD‐1 therapy enhanced immune activation in ovarian cancer patients, thereby improving survival.

## 2 Results

### 2.1 EVs from Ovarian Cancer Cells Exhibit High Levels of SPHK1

SPHK1 is a cytoplasmic protein, but a previous report suggested that it is secreted into the culture medium of tumor cells.^[^ ^9^ ^]^ However, the exact mechanism of its release and how that is modulating the tumor microenvironment to support tumor growth are unclear. Thus, we sought to determine if EVs release SPHK1 into the tumor microenvironment. To determine SPHK1 expression levels in ovarian cancer EVs, we first isolated and characterized EVs from culture media of ovarian cancer cells as described before.^[^ ^10^ ^]^ The isolated EVs were then analyzed by transmission electron microscopy (TEM) and nanoparticle tracking analyzer, which confirmed them to be ≈50–200 nm in size with an average size of 150 nm (Figure S1A,B, Supporting Information). We also confirmed the isolation of EVs by positive and negative markers detection on the western blot (Figure S1C, Supporting Information). We confirmed the quality and enrichment of the EVs in our preparations by using sucrose gradient centrifugation.^[^ ^11^ ^]^ Consistent with known EV fractionation protocols,^[^ ^12^ ^]^ fractions 4 and 5 (of 8), representing the 20–40% sucrose gradient, contained the highest levels of EV markers (CD63 and ALG‐2‐interacting protein‐X, ALIX) and SPHK1 (Figure S1D, Supporting Information). Therefore, we selected those fractions for all our future experiments.

**Figure 1:**
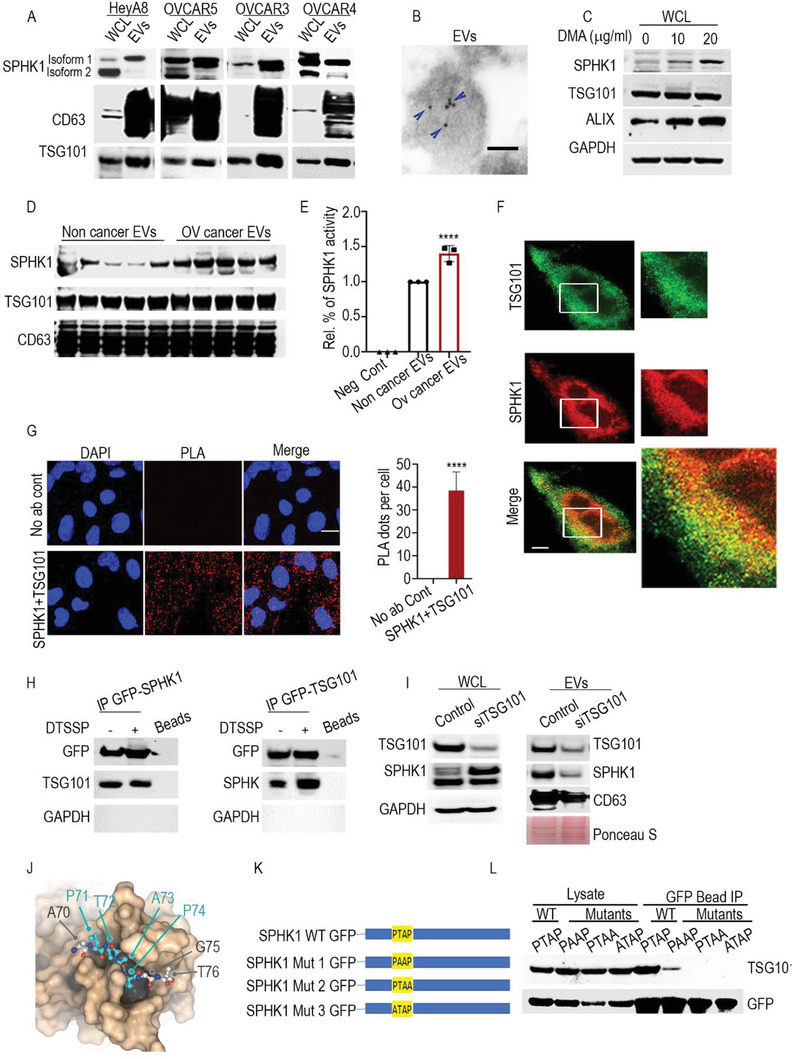
Secretion of SPHK1 in tumor associated EVs. A) Immunoblots for SPHK1 in whole cell lysate (WCL) and purified EVs from the indicated metastatic ovarian cancer cell lines. All lanes were loaded with the same amount of total protein. B) Representative TEM image of EVs (derived from HeyA8 cells) immunogold‐labeled with anti‐SPHK1 antibodies. Arrowheads indicate 10 nm gold particles in EVs. Scale bar, 50 nm. C) Western blot analysis of HeyA8 whole cell lysate (WCL) treated with DMA. D) Western blot analysis of SPHK1 expression in EVs isolated from plasma of healthy controls and ovarian cancer patients. E) SPHK1 activity was measured in EVs isolated from plasma of healthy controls (*N* = 3) and ovarian cancer patients (*N* = 3). Mean of relative % of SPHK1 activity is shown with standard error of mean (SEM) in comparison to EVs from the control group. ****p < 0.0001 (one‐way analysis of variance {ANOVA}). F) SPHK1 colocalization with TSG101 in HeyA8 cells. SPHK1 was stained with STAR RED and TSG101 was stained with STAR GREEN. Images were taken by an STED microscope with 100xSTED objective and 4.55 zoom. Image analysis was performed by Image J software, using JACoP plugin. G) Physical closeness of SPHK1 and TSG101 was evaluated by an in situ proximity ligation assay (PLA). Bar graph showing mean of red PLA dots with SEM counted manually in cells/3 fields. ****p < 0.0001 (Student's *t*‐test). H) Coimmunoprecipitation of GFP‐SPHK1 and GFP‐TSG101 fusion proteins, their binding partners with GFP‐beads, and detection by immunoblotting. I) Immunoblotting for whole cell lysate (WCL) and EVs from HeyA8 cells after TSG101‐siRNA treatment. J) Structural basis for SPHK1 PTAP motif recognition by the TSG101 Ubiquitin E2 variant (UEV) domain (model based on protein data bank (PDB) ID 1M4Q). SPHK1 residues 70–76 (ball‐and‐stick) are expected to occupy a deep cleft on the UEV domain surface. K) Sequence of binding motif in wild‐type SPHK1 and mutated SPHK1. L) The PTAP domain of SPHK1 is responsible for the interaction with TSG101. In vitro pulldown assay was performed on recombinant wild‐type SPHK1 and mutated SPHK1 (PTAP → PAAP, PTAA, or ATAP) and the mutated proteins’ interactions with TSG101 were determined by western blotting.

Immunoblotting of whole cell lysates (WCLs) and EVs isolated from four ovarian cancer cell lines (HeyA8, OVCAR5, NIH‐OVCAR3, and OVCAR4) demonstrated that SPHK1 isoform 2 was highly expressed in the secreted EVs compared to the WCLs of all four cell lines (**Figure** **1**A). Next, we analyzed the expression of SPHK1 in EVs by immunoelectron microscopy. This technique provides ultrastructural information and molecular localization (Figure 1B), we found 10 nm gold‐labeled SPHK1 within the EVs.

Conversely, treatment with 5‐(*N*,*N*‐dimethyl) amiloride hydrochloride (DMA, an inhibitor of EV biogenesis)^[^ ^13^ ^]^ increased SPHK1 levels in WCLs (Figure 1C), suggesting that blocking EV biogenesis inhibits the packaging of SPHK1 inside EVs, causing intracellular levels to rise. In a complementary approach, we generated a GFP‐tagged SPHK1 ectopic expression plasmid, which was transfected into HeyA8 cells and identified by the presence of green fluorescent protein (GFP) in EVs. This again confirmed that SPHK1 had been loaded into EVs (Figure S1E, Supporting Information).

We next examined the amount of SPHK1 released in EVs isolated from blood samples collected from women with ovarian cancer and healthy controls. We found higher levels of SPHK1 in EVs secreted by ovarian cancer patients than in those secreted by healthy controls (Figure 1D). Importantly, our sphingosine kinase activity assay demonstrated that SPHK1 in the EVs isolated from human plasma was enzymatically active compared to the negative controls. We also found that SPHK1 in the EVs of cancer patients was highly active compared with that from the healthy controls as well as from cells lines HeyA8 and OVCAR5 (Figure 1E and Figure S1F,G, Supporting Information). To investigate whether EVs can produce S1P independent of cells, we isolated HeyA8 cells EVs by ultracentrifugation and incubated 100 µg of EVs in 2 mL of culture media for 24 h and quantified S1P released in culture media by mass spectrometry. We were able to quantify 20.49 pg S1P mL^−1^ of media. These results suggested that EVs are able to synthesize S1P as an independent unit (Figure S1H, Supporting Information) and sphingosine is one of the component of EVs membrane.^[^ ^14^ ^]^ Compatible with the above finding, our data suggest that SPHK1 in EVs convert sphingosine to S1P in the EVs membrane, which leads to the bursting of EVs and releasing of S1P in the extracellular space.

The task of sorting cargo into EVs involves specific proteins associated with the endosomal sorting complex required for transport, such as ALIX and tumor susceptibility gene 101 protein (TSG101).^[^ ^15^ ^]^ Therefore, we next sought to determine if SPHK1 directly interacts with any key protein component of EVs, such as TSG101.

We validated these findings in vitro by determining the interaction of SPHK1 with TSG101. HeyA8 cells were stained with antibodies directed against SPHK1 and TSG101, and their positions were visualized using stimulated emission depletion (STED) microscopy. This showed that SPHK1 strongly colocalized with TSG101 (Mander's colocalization coefficient value = 0.98), further supporting the notion that the two interact (Figure 1F). That interaction was further verified by a proximity ligation assay (PLA), which revealed high levels of proximity ligation signals when we used target‐specific antibodies against SPHK1 and TSG101 (Figure 1G).

To further validate this binding, we used GFP‐Trap beads to immunoprecipitate GFP‐tagged SPHK1 from cancer cells and we found that SPHK1 does indeed interact with TSG101. Adding a nonspecific protein crosslinker, 3,3'‐dithiobis sulfosuccinimidyl propionate (DTSSP), stabilized the interaction. In a complementary approach, we found that HeyA8 cells expressing TSG101‐GFP coimmunoprecipitated with SPHK1, again revealing that TSG101 interacts with SPHK1 (Figure 1H). To further confirm this unique interaction, we used TSG101 small interfering RNA (siRNA) to block the expression of TSG101 in HeyA8 cells and analyzed the release of SPHK1 in EVs. As shown by western blotting (Figure 1I), silencing TSG101 in cells increased the expression of SPHK1 in WCLs. However, it significantly reduced the expression of SPHK1 in EVs, indicating that TSG101 is an important mediator of the release of SPHK1 into EVs.

Comparison of the sequences of SPHK1 isoforms 1 and 2 revealed an N‐terminal extension in isoform 2 that also contains a PTAP motif, which is known to bind the UEV domain of TSG101 (Figure 1J). Based on published structure‐function data for TSG101,^[^ ^16^ ^]^ we speculated that the PTAP motif in SPHK1 isoform 2 is required for interaction with the EVs component TSG101. To test this hypothesis, we mutated the PTAP motif into PAAP, PTAA, or ATAP (Figure 1K). Then the three mutated plasmids and the wild type (WT) SPHK1 were transfected into HeyA8 cells. We then performed pulldowns using a GFP‐Trap. Importantly, we found that the PTAA and ATAP mutations abrogated the interaction of SPHK1 with TSG101, whereas the PAAP mutation only partially inhibited that interaction. Taken together, our data prove that the PTAP motif is important for the binding of SPHK1 to TSG101 (Figure 1L), and they demonstrate that SPHK1 is packaged into EVs because it can interact with TSG101 (Figure S1I, Supporting Information).

### 2.2 S1P Signaling Induces PD‐L1 Expression on Tumor Cells

**Figure 2:**
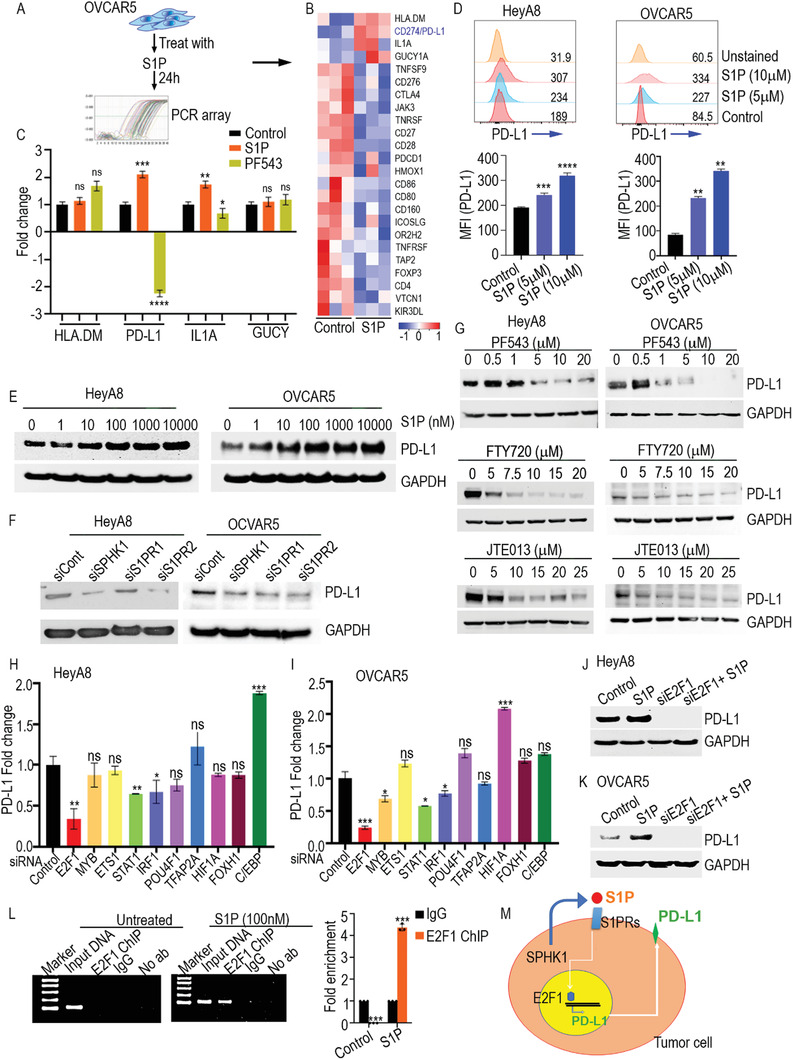
Regulation of expression of PD‐L1 on tumor cells by S1P. A,B) Human immune checkpoint qPCR array was performed using complementary DNA (cDNA) prepared from OVCAR5 cells that were stimulated with S1P (100 × 10^−9^ m). Gene expression is represented as log2 of Ct values, with *p* < 0.05 as compared to untreated control. Genes that were ≥1.5‐fold downregulated (blue) or upregulated (red) were included in the heat map. Results were analyzed by one‐way ANOVA in comparison to control. **p < 0.01, ***p < 0.001, ****p < 0.0001. C) A qPCR array was used to validate upregulated genes in OVCAR5 cells. Results are presented as mean fold change with SD in comparison to control. *p < 0.05, **p < 0.01, ***p < 0.001, ****p < 0.0001 (one‐way ANOVA). D) Flow cytometric quantification of surface expression of PD‐L1 in HeyA8 and OVCAR5 cells after stimulation with different doses of S1P. The numerical values within the fluorescence activated cell sorting (FACS) overlay plots indicate mean fluorescent intensity (MFI). Adjacent bar diagrams represent cumulative data from three experiments. **p < 0.01, ***p < 0.001, ****p < 0.0001 (one‐way ANOVA). E) Ovarian cancer cell lines HeyA8 and OVCAR5 were stimulated with the indicated doses of S1P for 24 h and PD‐L1 expression was detected by western blotting. F) Ovarian cancer cell lines HeyA8 and OVCAR5 were transfected with siRNA of SPHK1, S1PR1, and S1PR2, and change in the expression of PD‐L1 was detected by western blotting. G) HeyA8 and OVCAR5 cells were treated with the S1P signaling inhibitors PF543 (SPHK1), FTY720 (S1PR1), or JTE013 (S1PR2) for 24 h at the indicated doses, and changes in PD‐L1 expression were detected by western blotting. H,I) Validation of the transcription factor array assay by qPCR in HeyA8 and OVACR5 cells lines after treatment with the respective transcription factor siRNAs. Results are presented as mean fold change with standard deviation (SD) in comparison to control. *p < 0.05, **p < 0.01, ***p < 0.001 (one‐way ANOVA). J,K) HeyA8 and OVACR5 cells were stimulated with S1P (5 × 10^−6^ m) or S1P in combination with E2F1 siRNA, and expression of PD‐L1 was determined by western blotting. L) HeyA8 cells were stimulated with S1P (100 × 10^−9^ m) for 24 h or left untreated. A ChIP assay was performed with E2F1 antibody; isotype IgG was used as the control. A sample without antibody was used to avoid unspecific binding to the agarose beads used to perform the ChIP. PCR product was run on agarose gels, and a 100 bp amplicon was found in the input DNA and E2F1 ChIP sample after S1P stimulation, whereas only the input DNA was amplified in the control cells. The bar graph shows the qPCR reaction analyzed by fold enrichment. ***p < 0.001 (Student's *t*‐test). M) Schematic presentation of S1P signaling regulating the expression of PD‐L1 via E2F1.

Based on our data, we also hypothesized that SPHK1 in extracellular vesicles promotes the extracellular maturation of S1P. Although S1P is a known regulator of immune cell trafficking,^[^ ^5^ ^]^ the signaling mechanism of how it suppresses the immune system in cancer is not fully known. Therefore, we created a quantitative polymerase chain reaction (qPCR) array containing 86 genes that included both immune checkpoints and immune cell modulators. Total RNA was isolated from OVCAR5 cells treated with 100 × 10^−9^ m S1P or with the SPHK1 inhibitor PF543 for 24 h. This approach revealed that S1P stimulation had modified several genes associated with immune checkpoints, whereas PF543 had inhibited the expression of several sets of genes (**Figure** **2**A,B and Figure S2A and Table S1, Supporting Information). PD‐L1 (also known as CD274) showed the most prominent change, as its expression increased more than twofold, followed by IL1A. The expression of both genes was inhibited by PF543 (Figure 2C). We also observed that S1P treatment in tumor cells inhibited the expression of TAP2, CD27, CD160, and ICOSLG genes, which are important for the immune cell activation needed for antitumor responses (Figure 2B). To decide whether the changes we identified might be generalizable in ovarian cancer, we treated two additional ovarian cancer cell lines (HeyA8 and OVCAR4) with S1P (100 × 10^−9^ m) or PF543 (10 × 10^−6^ m). Again, we found that PD‐L1 expression was upregulated by S1P stimulation. Conversely, PF543 reduced PD‐L1 expression in both ovarian cancer cell lines (Figure S2B,C, Supporting Information).

Epithelial ovarian cancer is not responsive to immunotherapy treatment, and the exact mechanism underlying this problem is not well understood. Given that PD‐L1 is an important regulator for weakening T cell responses in cancers, we focused on how S1P promotes PD‐L1 expression. First, we confirmed that S1P promotes PD‐L1 expression on ovarian cancer cells using flow cytometry and immunoblotting. As expected, S1P stimulation‐induced PD‐L1 protein levels dose‐dependently (Figure 2D,E and Figure S2D,E, Supporting Information).

In a complementary approach, we used siRNAs specific to SPHK1, S1PR1, and S1PR2 to knock down those proteins’ expression in ovarian cancer cells. Loss of SPHK1, S1PR1, and S1PR2 reduced PD‐L1 expression (Figure 2F). We also used the chemical inhibitors PF543, FTY720, and JTE013, which inhibit SPHK1, S1PR1, and S1PR2, respectively. All three inhibited the expression of PD‐L1 in ovarian cancer cells (Figure 2G). Additionally, PF543 reduced the expression of PD‐L1 in murine ID8 Trp53^−/−^; Brca2^−/−^ cells (Figure S2F, Supporting Information).

To determine if S1P regulates the transcription of PD‐L1, we cloned the PD‐L1‐promotor upstream to the luciferase reporter and transfected the construct into OVCAR5 cells. We then stimulated those cells with S1P or treated them with inhibitors and checked for luciferase activity. As expected, S1P increased PD‐L1 promoter activity compared to control, while the S1PR1, S1PR2, and SPHK1 inhibitors reduced PD‐L1 promoter activity (Figure S2G,H, Supporting Information), demonstrating that S1P activates PD‐L1 transcription.

To identify specific transcription factors (TFs) required for S1P‐mediated PD‐L1 transcription and are critical for PD‐L1 expression, we stimulated OVCAR5 cells with S1P, collected nuclear extracts, and performed a transcription factor profiling assay. S1P stimulation activated AP2, GR/PR, E2F1, HIF, EGR, POU4F1, ER, c/EBP, ETS‐1, FOXH1, and MYB transcription factors (Figure S2I, Supporting Information). To confirm the involvement of these transcription factors, we knocked them down in ovarian cancer cell lines, using target‐specific siRNAs, and performed qPCR to measure PD‐L1 expression. Loss of E2F1 reduced PD‐L1 expression, which could no longer be induced by S1P stimulation (Figure 2H–K). Next, we sought to determine whether E2F1 binds directly onto the PD‐L1 promoter and identify which sequences in PD‐L1 are critical for binding. We used MatInspector TF prediction software for this analysis, which identified several consensus sequences in E2F1 and the PD‐L1 promoter with >1 matrix consensus scores (Table S2, Supporting Information). Next, we performed a chromatin immunoprecipitation (ChIP) assay to confirm the binding of E2F1 with the PD‐L1 promoter, treating predicted sequences in HeyA8 cells with or without S1P. Importantly, S1P treatment enriched PD‐L1 promoter sequences when E2F1 was immunoprecipitated (compared to control IgG) (Figure 2L). Taken together, our data show that S1P signaling causes immune suppression through E2F‐1‐mediated PD‐L1 upregulation (Figure 2M).

To further determine how SPHK1 upregulates PD‐L1 and how it affects tumor growth in vivo, we ectopically expressed (EE) SPHK1 in HeyA8 cells (Figure S2J, Supporting Information) and orthotopically injected those cells or control cells into the ovarian bursa of nude mice and monitored tumor growth. SPHK1 markedly increased tumor growth at the primary injection site and distant organs in the peritoneal cavity (Figure S2K–M, Supporting Information). Total tumor weight and the number of tumor nodules increased when we ectopically expressed SPHK1 compared to control (Figure S2N,O, Supporting Information). Immunoblot analysis of the tumor lysates also showed that tumors with high SPHK1 expressed high levels of both SPHK1 and PD‐L1 compared to their controls (Figure S2P, Supporting Information). Immunohistochemistry of the tumor tissues demonstrated that the ectopically expressed SPHK1 group had increased levels of the proliferation marker Ki67 and both SPHK1 and PD‐L1 (Figure S2Q, Supporting Information). Taken together, our data show that high expression of SPHK1 increased both tumor burden and expression of PD‐L1 in ovarian cancer in vivo.

### 2.3 EVs Encapsulating SPHK1 Cause Immune Suppression in Ovarian Cancer

**Figure 3:**
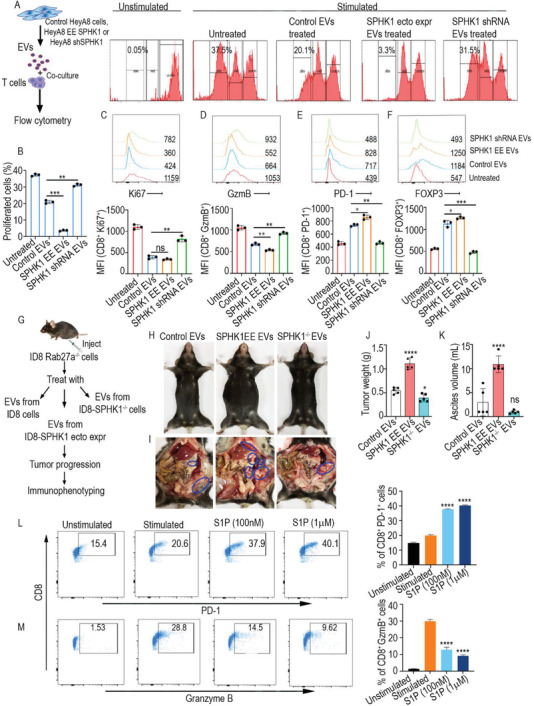
Suppression of T cells cytotoxicity by EVs associated SPHK1. A) Treatment of T cells purified from human blood with EVs isolated from control HeyA8 cells, from ectopically expressed SPHK1, or from cells knocked down with SPHK1 ShRNA. CFSE‐stained CD8 T cells were treated with the indicated EV types and stimulated with anti‐CD3 and anti‐CD28 antibodies. Histograms were generated by flow cytometry. B) CFSE staining showed the percentage of T cells that proliferated with the indicated treatment. The results were analyzed with FlowJo software and mean % of proliferative cells were plotted as bar graphs. **p < 0.01, ***p < 0.001 (one‐way ANOVA). C) Ki67 staining was performed and MFIs were calculated by flow cytometry after purified human blood cells received the indicated treatments with EVs. D) Granzyme B staining was performed, and MFIs were calculated by flow cytometry after human T cells received the indicated treatment with EVs. E) PD‐1 staining was performed and MFIs were calculated by flow cytometry after human T cells received the indicated treatment with EVs. F) FOXP3 staining was performed and MFIs were calculated by flow cytometry after the indicated EV treatments of human T cells flow cytometry after human T cells received the indicated treatments with EVs. Histograms were plotted by FlowJo software indicated MFI values. Mean MFI were plotted as bar graphs with SEM. *p < 0.05, **p < 0.01, ***p < 0.001 (one‐way ANOVA). G) Schema showing implantation of RAB27a^−/−^ ID8 cells into the ovary of C57BL/6 mice and subsequent treatment with the indicated EVs (*N* = 5 per group). H) General appearance of mice at the end of treatment. The mice treated with EVs carrying ectopically expressed SPHK1 had a greater ascites volume, whereas the mice treated with control EVs that did not express SPHK1 appeared normal. I) Peritoneal cavity of mice showing tumor locations (blue circles) in all treatment groups. J) Bar graph indicates average tumor weight and K) average ascites volume among the EV‐treated mice. L,M) Human blood CD8 T cells were stimulated with S1P, and PD‐1 and granzyme B were measured by flow cytometry. Percentages of CD8+ PD‐1+ cells and CD8+ granzyme B+ cells were determined by FlowJo. All results are shown as the mean of three technical replicates ± SEM. **p < 0.01, ***p < 0.001, ****p < 0.0001 compared to control by one‐way ANOVA.

We hypothesized that EVs facilitate the transport of SPHK1 from the cytoplasm to extracellular space to promote the maturation of S1P and create an immunosuppressive microenvironment in tumors. To determine how SPHK1 packaged in EVs affects T cell proliferation, we isolated human CD8+ T cells from healthy human blood and labeled them with CFSE (carboxyfluorescein succinimidyl ester), a cell tracker dye. The labeled CD8+ T cells were then stimulated with CD3 and CD28 antibodies and coincubated for 3 d with EVs isolated from i) control HeyA8 cells, ii) HeyA8 cells that ectopically expressed SPHK1, or iii) HeyA8 cells silenced SPHK1‐shRNA. Flow cytometry showed that treating EVs that expressed SPHK1 decreased the proliferation of the human CD8+ T cells compared to control EVs. In contrast, we observed that T cell proliferation increased after treatment with EVs collected from cells that expressed SPHK1‐shRNA (**Figure** **3**A,B). In conjunction, we found higher Ki67 and granzyme B expression in CD8+ T cells treated with EVs collected from SPHK1‐shRNA‐expressing cells than CD8+ T cells treated with control EVs. Conversely, treatment with EVs collected from SPHK1‐ ectopically expressing cells decreased levels of Ki67 and granzyme B in CD8+ T cells compared to the control (Figure 3C,D). We also observed that PD‐1 and FOXP3 expression were higher when T cells were treated with EVs expressing SPHK1 compared to their respective control EVs (Figure 3E,F). Taken together, our data demonstrate that SPHK1‐packaged EVs significantly contribute to the regulation of T cell activation and proliferation.

Next, we investigated if SPHK1‐packaged EVs cause immune suppression along with increased tumor growth and metastasis in vivo. In brief, we injected RAB27a^−/−^ ID8 cells into the ovary of C57BL/6 mice. The resulting tumors could express SPHK1 but could not release EVs because the RAB27a gene necessary for vesicle trafficking was knocked out. Ten days after the tumor cells were injected, the mice were randomized and treated with EVs isolated from i) parental ID8 cells, ii) ID8 cells that EE SPHK1, or (iii) SPHK1^−/−^ ID8 cells (Figure 3G). The animals were then monitored for tumor growth. In this experiment, we found that delivery of EVs collected from cells that ectopically expressed SPHK1 increased tumor weight, ascites volume, and body weight compared with EVs isolated from parental cells (Figure 3H–K and Figure S3A, Supporting Information). We found that tumor growth was slower when mice were injected with EVs isolated from SPHK1^−/−^ cells compared to EVs collected from WT cells (Figure 3J,K). However, we saw no significant change in ascites volume. Immunophenotyping of T cells in ascites from EVs‐treated mice demonstrated an increased number of CD8 and CD4 positive T lymphocytes (TILs) in the SPHK1^−/−^ EVs group compared to the control EVs group, while the SPHK1 EE EVs group showed significantly less infiltration of CD8 and CD4 cells into ascites compared to control (Figure S3B,C, Supporting Information). Expression of proliferation marker Ki67 and T cell activation markers‐granzyme B and Tumor Necrosis Factor (TNF) alpha was also higher in both CD8 and CD4 positive TILs in the SPHK1^−/−^ EVs treated mice compared to the control EVs treated animals, while SPHK1 EE EVs did not show any significant change in these markers compared to control EVs (Figure S3D–I, Supporting Information). Similarly, we found reduced expression of the T cell exhaustion markers PD‐1 and TIM3 in TILs isolated from SPHK1^−/−^ EVs treated ascites than in control EVs group, while SPHK1 EE EVs treated mice showed higher expression of these markers compared to control (Figure S3J–M, Supporting Information). Furthermore, the percentage of FOXP3+ T regulatory cells was reduced in the SPHK1^−/−^ EVs ‐treated group compared to the control mice (Figure S3N,O, Supporting Information). To further confirm that tumor‐associated EVs loaded with SPHK1 promote ovarian cancer progression, we performed a similar in vivo experiment by injecting SPHK1^−/−^ ID8 cells treated with EVs isolated from parental ID8 cells or ID8 cells that ectopically expressed SPHK1 (Figure S4A, Supporting Information). As expected, tumor weight, ascites volume, and body weight drastically increased in the mice treated with EVs containing ectopically expressed SPHK1 compared to the group treated with EVs from parental cells (Figure S4B–F, Supporting Information). Additionally, we injected parental ID8 cells, RAB27a^−/−^ ID8 cells, SPHK1^−/−^ ID8 cells, and SPHK1 ectopic expressing (EE) ID8 cells in the ovary of C57BL/6 mice to know the direct association of SPHK1 with the ovarian tumor growth. We saw both RAB27a^−/−^ ID8 cells, SPHK1^−/−^ ID8 cells made less solid tumor ascites in mice compared to both parental ID8 cells as well as SPHK1 EE ID8 cells (Figure S4G–K, Supporting Information). Thus, our data show that SPHK1, associated with EVs, has a critical role in ovarian tumor progression.

Next, we hypothesized that SPHK1‐packaged EVs can increase the concentration of S1P in the tumor microenvironment, further inducing immune suppression. To confirm that S1P signaling is involved in T cell suppression, we treated purified human CD8 T cells with increasing concentrations of S1P, finding that S1P treatment increased the expression of PD‐1 (Figure 3L). In contrast, S1P decreased the expression of granzyme B and Ki67 (Figure 3M and Figure S5A, Supporting Information). Next, we performed a cytokine bead array with culture medium from human CD8 T cells treated with EVs isolated from i) control HeyA8 cells, ii) HeyA8 cells that ectopically expressed SPHK1, or iii) HeyA8 cells silenced SPHK1‐shRNA. The results showed that immune‐activating cytokines such as IL‐17, Interferon gamma (IFN*γ)*, and TNF alpha were suppressed upon control EVs or SPHK1 EE EVs treatment while SPHK1 shRNA EVs could not impact the expression of these cytokines compared to untreated T cells (Figure S5B–D, Supporting Information). Secretion of immunosuppressive cytokines such as IL‐6, IL‐10, and IL‐4 reduced upon SPHK1 shRNA EVs treatment compared to control EVs or SPHK1 EE EVs treatment (Figure S5E–G, Supporting Information). We also performed a similar cytokine bead array with culture medium from human CD8 T cells treated S1P. The array showed that immune‐activating cytokines such as IL‐17, IFN*γ*, and TNF alpha were suppressed while secretion of the immunosuppressive cytokine IL‐6 increased (Figure S5H–K, Supporting Information). However, secretion of other immunosuppressive cytokines, such as IL‐10 and IL‐4, was decreased (Figure S5L–M, Supporting Information). Overall, a high expression of S1P suppresses the cytotoxic behavior of T cells in the tumor microenvironment.

To further confirm the S1P‐mediated reduction in T cell cytotoxicity, we isolated immature DCs from normal C57BL/6 mice bone marrow and incubated them with ID8 cell lysate for maturation. To activate naive CD8 T cells isolated from normal mice spleen, we cocultured them with mature DCs in the presence or absence of S1P stimulation. We then incubated ID8 tumor cells with the activated CD8 T cells and quantified cell death with Annexin V Red (IncuCyte). Importantly, we found that S1P stimulation reduced the cytotoxicity of T cells as determined by counting the cell death of ID8 cells (Figure S5N–P, Supporting Information). We also observed reduced levels of Ki67 and granzyme B expression in the S1P‐treated T cells (Figure S5Q,R, Supporting Information), while expression of PD‐1 increased (Figure S5S, Supporting Information). Overall, these data show that SPHK1 loaded into EVs can increase S1P levels in the tumor microenvironment, further promoting immune suppression.

### 2.4 Systemic Inhibition of SPHK1 by PF543 Increases T Cell Proliferation and Activity In Vivo

**Figure 4:**
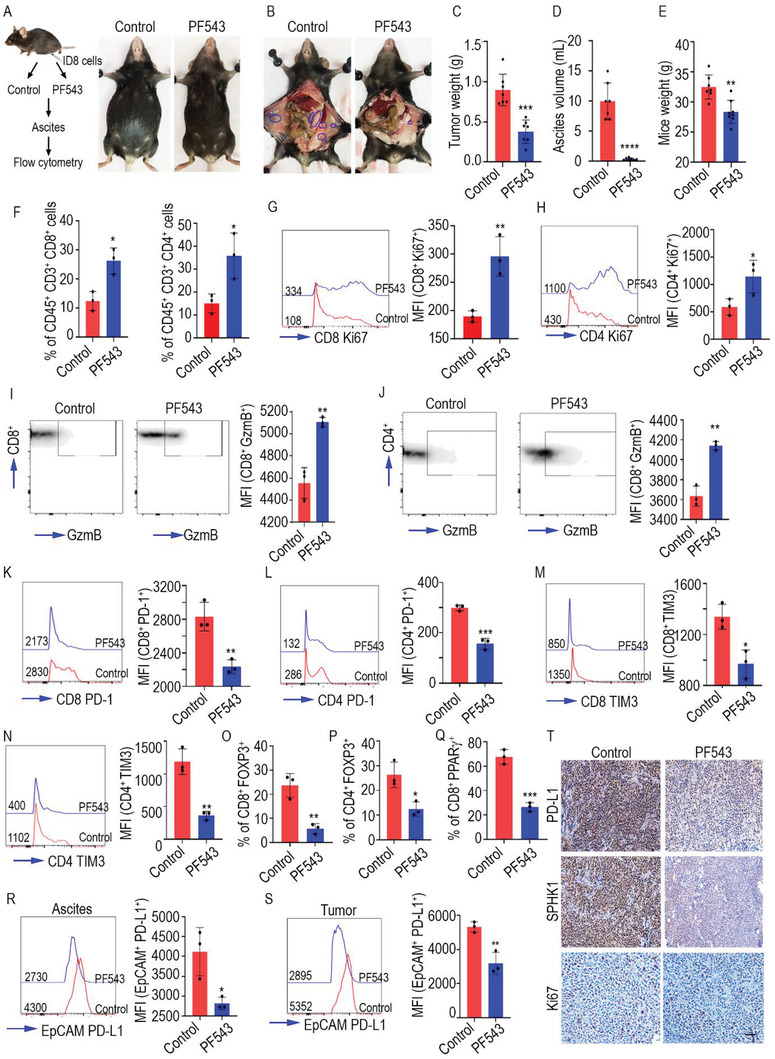
SPHK1 inhibitor PF543 reduced ovarian cancer progression via increasing T cell cytotoxicity. A) Schema showing WT mice C57BL/6 mice orthotopically inoculated with ID8 cells in the right ovary. The mice were grouped as control (*N* = 7) and PF543‐treated (*N* = 7). At the end of treatment, the control mice had greater volume of ascites, whereas the PF543‐treated mice appeared normal. B) Peritoneal cavity of mice showing tumor locations (blue circles) in control mice versus mice treated with PF543. C) Differences in average tumor weight D), ascites volume, and E) body weight between control and PF543 treated mice. F) Flow cytometric quantification of the percentages of CD8+ and CD4+ cells among CD45+ and CD3+ cells in ascites 82 d after the mice (*N* = 3 per group) were injected with tumor cells. Quantification of Ki67+ cells among G) CD8+ and H) CD4+ T cells in ascites (*N* = 3 per group). Quantification of Granzyme B+ cells among I) CD8+ cells and J) CD4+ T cells in ascites (*N* = 3 per group). Quantification of PD‐1+ cells among K) CD8+ cells and L) CD4+ T cells in ascites (*N* = 3 per group). Quantification of TIM3+ cells among M) CD8+ cells and N) CD4+ T cells in ascites (*N* = 3 per group). Percentages of FOXP3+ cells, O) CD8+ cells, and P) CD4+ T cells among CD45+ and CD3+ cells in ascites of mice (*N* = 3 per group). Q) Quantification of the percentages of PPARg+ cells among CD8+ T cells in the ascites of mice (*N* = 3 per group). Quantification of PD‐L1+ cells among EpCAM+ tumor cells in R) ascites and S) tumor tissue in mice (*N* = 3 per group). Numerical values within the FACS overlay plots indicate mean fluorescent intensity (MFI). Adjacent bar diagrams represent cumulative data from three experiments. *p < 0.05, **p < 0.01, ***p < 0.001, ****p < 0.0001 (Student's *t*‐test). T) Immunohistochemical analysis of tumor tissues selected from each group (*N* = 3) was performed with the indicated antibodies. Positivity is shown as brown staining of 3, 3'‐diaminobenzidine (DAB).

PF543 is the most potent inhibitor of SPHK1 known to date and it can be used for in vivo studies.^[^ ^17^ ^]^ To determine the effects of pharmacological inhibition of SPHK1 on tumor growth and T cell activation, we orthotopically injected ID8 cells into C57BL/6 syngeneic mice and treated the animals with PF543 (ten mice per group). We terminated the experiment when control mice became moribund. Notably, PF543 treatment reduced tumor weight, ascites volume, and body weight compared to the control (**Figure** **4**A–E). Immunophenotyping of T cells in ascites from control and PF543‐treated mice demonstrated an increased number of CD8 and CD4 positive T lymphocytes (TILs) in the PF543 group (Figure 4F). Ki67 and granzyme B expression was also higher in both CD8 and CD4 positive TILs in the PF543 treated mice compared to the untreated animals (Figure 4G–J). Similarly, we found reduced expression of the T cell exhaustion markers PD‐1 and TIM3 in TILs isolated from PF534 treated ascites than in the control group (Figure 4K–N). Furthermore, the percentage of FOXP3+ T regulatory cells was reduced in the PF543‐treated group compared to the control mice (Figure 4O,P). Recently, it has been found that targeting SPHK1 in T cells activates T cells cytotoxicity via Peroxisome proliferator‐activated receptor gamma (PPAR*γ)* downregulation,^[^ ^2^ ^]^ we also checked the expression of PPAR*γ* by flow cytometry after PF543 treatment, finding that its expression was relatively less in the PF543‐treated mice (Figure 4Q). We further analyzed the expression of transcription factor transcription factor T cell factor 1 (TCF‐1) in TILs after PF543 treatment. TCF‐1 is a critical regulator of T‐cell development, and self‐renewing progenitors T cells are positive for TCF‐1.^[^ ^18^ ^]^PF‐543 treatment was found to increase the expression of TCF‐1 on both CD8 and CD4 positive TILs. Thus, it is showing that PF543 can enhance the self‐renewing properties of T cells in ascites (Figure S6A,B, Supporting Information).

To explore the effect of PF543 on PD‐L1 expression in vivo, we compared expression in tumor cells in both ascites and solid tumors from PF543‐treated mice versus untreated mice. As shown in Figure 4R,S, PD‐L1 expression was decreased in EpCAM‐positive tumor cells from ascites and ovarian tumors in the PF543‐treated mice compared to the controls. Our IHC also showed a marked decrease in SPHK1, PD‐L1, and Ki67 levels in tumor cells after PF543 treatment (Figure 4T). To determine whether PF543 can reduce the extracellular S1P levels in the tumor microenvironment, we performed S1P staining by IHC and found reduced S1P levels in PF543 treated tumor tissues compared to control (Figure S6C, Supporting Information). Overall, our data demonstrate that systemic inhibition of SPHK1 reduced both S1P‐mediated signaling and ovarian cancer growth by activating both CD4 and CD8 positive T cells.

As S1P is known to regulate T cell trafficking,^[^ ^5^ ^]^ we sought to determine the effect of PF543 treatment on T cell migration in various organ tissues in normal and tumor‐bearing C57BL/6 mice. CD8 and CD4 populations were significantly greater in the spleen (Figure S6D, Supporting Information), and lymph nodes (Figure S6E, Supporting Information) of the PF543 treated mice than untreated controls. However, we saw no change in T cell infiltration in other organs such as bone marrow, liver, and lung (Figure S6F–H, Supporting Information). Notably, the T cell population of the PF543‐treated normal mice was also unaffected in various organs such as bone marrow, liver, lung, lymph node, spleen, and blood compared to untreated normal mice (Figure S6I–N, Supporting Information) as shown in Figure S6O in the Supporting Information gating strategy. Our data demonstrate that systemic treatment with PF543 did not affect T cell migration and trafficking adversely.

### 2.5 Combining SPHK1 Inhibitor with PD‐1‐Blocking Antibody Reduces Ovarian Cancer Growth and Progression, Improving Survival

**Figure 5:**
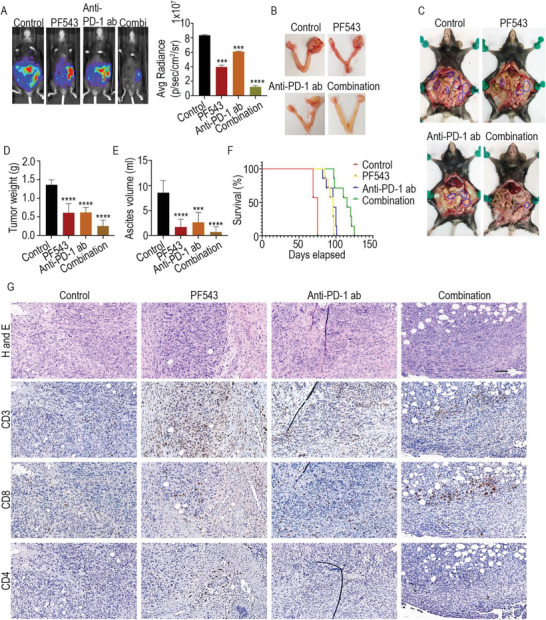
Combination of PF543 synergies with anti‐PD‐1 therapy to reduce ovarian cancer burden. A) C57BL/6 mice were orthotopically injected with ID8 cells in the right ovary. Mice were grouped as control (*N* = 10), PF543‐ treated (*N* = 10), anti‐PD‐1 antibody‐treated (*N* = 10), and combination‐treated (PF543 + anti‐PD‐1 antibody) (*N* = 10). Mice from all groups were imaged using biophotonic in vivo imaging system (IVIS), and representative photographs were presented. The bar graph indicates the average radiance of luminescence intensity. ***p < 0.001, ****p < 0.0001 compared to control by one‐way ANOVA. B) Appearance of ovaries with the indicated treatments. C) Peritoneal cavity of mice showing tumor locations (blue circles) in control versus treatments. The bar graph indicates D) average tumor weight and E) average ascites volume in control versus treated mice. ***p < 0.001, ****p < 0.0001 compared to control by one‐way ANOVA. F) Survival curve of control (*N* = 7) and treated (*N* = 7) mice injected with ID8 Trp53^−/−^; Brca2^−/−^ intraperitoneally. G) IHC staining of tumor tissues from the control, PF543, anti‐PD‐1 antibody, or combination (PF543 + anti‐PD‐1) groups. DAB staining (brown) was shown for the T cell markers CD3+, CD4+, and CD8+. Corresponding H&E‐stained sections were also shown. 20X images were obtained with CaseViewer software.

Given the effect of SPHK1 inhibitor on tumor cells and T cells, we hypothesized that administering PF543 along with anti‐PD‐1 antibody should improve anti‐PD‐1 therapy for ovarian cancer by increasing the antitumor responses of T cells. Therefore, we injected ID8 cells into the ovary of C57BL/6 mice and randomized the animals into four groups: i) control, ii) with the SPHK1 inhibitor PF543, iii) with anti‐PD‐1 antibody, and iv) with PF543 + anti‐PD‐1 antibody (ten mice per group). Strikingly, the combination group (iv) had a significantly fewer tumor nodules and decreased ascites volume compared to the single‐treatment groups (ii and iii) (**Figure** **5**A–E). These data show that SPHK1 inhibition synergizes the blockade of PD‐1 by reducing ovarian cancer progression. To complement these observations, we observed that the combination of PF543 and anti‐PD‐1 antibody also improved the survival of mice bearing ovarian cancer compared to either single treatment (Figure 5F). Moreover, infiltration of CD3+ T cells into mouse tumor tissues treated with either PF543 or anti‐PD‐1 antibody was enhanced compared to untreated tumor tissues (Figure 5G) as detected by IHC staining of CD3, CD4, and CD8 markers. In the combination group, there was increased infiltration of CD8+ T cells.

### 2.6 SPHK1 Expression Associates with PD‐L1 Expression in Clinical Samples of Ovarian Cancer

**Figure 6:**
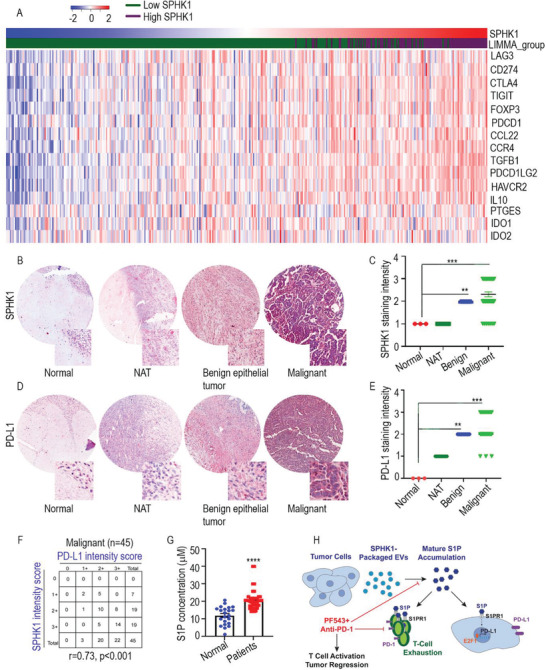
SPHK1 expression correlates with PD‐L1 in ovarian cancer clinical samples. A) Heat map showing differential expression of selected immunosuppressive genes between low (*n* = 282) and high SPHK1 (*n* = 94) expression groups, using RNA‐Seq gene expression data from TCGA ovarian cancer cohort. B–E) Red indicates high expression and blue indicates low expression relative to a gene's mean expression across all samples. IHC was performed for SPHK1 and PD‐L1 for tissue microarray analysis (TMA). Staining intensity was scored manually, using a scale of 0, 1, 2, and 3. **p < 0.01, ***p < 0.001 compared to normal by one‐way ANOVA. F) Analysis of SPHK1 and PD‐L1 coexpression based on scoring the TMA slides. G) S1P levels were measured in plasma of healthy samples (*N* = 22) and ovarian cancer samples (*N* = 40). **p < 0.01, ***p < 0.001 (Student *t*‐test). H) S1P/SPHK1 signaling mechanism in ovarian cancer, with therapeutic indications.

Using RNAseq data to analyze the cancer genome atlas (TCGA) human ovarian cancer gene expression, we explored the association of SPHK1 expression with genes involved in immunosuppression in ovarian cancer. Interestingly, high expression of SPHK1 was associated with increased expression of those genes (**Figure** **6**A and Figure S7A, Supporting Information). Using the same dataset, we also observed high levels of PD‐L1, PD‐1, FOXP3, and E2F1 in the ovarian cancer samples that expressed high levels of SPHK1 compared to samples that expressed low levels (Figure S7B–E, Supporting Information). Our gene set enrichment analysis based on SPHK1 expression also demonstrated a positive association of genes associated with epithelial to mesenchymal transition, inflammatory response, and Kirsten rat sarcoma 2 viral oncogene homolog (KRAS) signaling (Figure S7F, Supporting Information). Notably, these hallmarks have previously been demonstrated to associate with S1P signaling.^[^ ^19^, ^20^ ^]^

Tissue microarrays (TMAs) showed that both SPHK1 and PD‐L1 were highly expressed in malignant tumor tissue in comparison with normal ovarian tissue and normal adjacent tissue. We also found significantly higher levels of SPHK1 and PD‐L1 in malignant tissue than in benign tissue (Table S3, Supporting Information, and Figure 6B–E). We could not find any correlation of SPHK1 expression with neoplasm staging (Table S3, Supporting Information). Notably, SPHK1 IHC scores correlated directly with PD‐L1 expression in the TMA samples (Figure 6F). In line with the above results, S1P levels were markedly higher in plasma from ovarian cancer patients than in plasma from healthy donors (Figure 6G). Collectively, our data demonstrate that SPHK1‐packaged EVs mediates extracellular modulation of S1P signaling, promoting the growth of ovarian tumors via immune suppression (Figure 6H).

We also performed the IHC staining of S1P in the normal ovary as well as in tumor ovary sections from different subjects. Images (Figure S8A,B, Supporting Information) show the positive staining of S1P in tumor microenvironment while normal ovaries show lesser expression of S1P in stroma.

## 3 Discussion

Although EVs are known to alter the tumor microenvironment, the key mechanisms by which they make ovarian cancer less immunogenic are not well characterized. We found that trafficking SPHK1 in EVs from the cytoplasm to the extracellular space is a critical part of those mechanisms. Collectively, our data strongly support the hypothesis that SPHK1 colocalizes and associates with EV proteins, facilitating the extracellular production of S1P. In essence, our data identify EVs as vehicles of SPHK1 secretion. We also found that interrupting the interaction between TSG101 and SPHK1 affected the packaging of SPHK1 into EVs.

While the roles of nucleic acids such as miRNAs, lncRNAs, and DNA fragments in EVs are well characterized, it is less clear how proteins are encapsulated into EVs. Using molecular docking, immunoprecipitation, pulldown experiments, proximity ligation assays, and high‐resolution microscopy (including immune electron microscopy), we proved that the exosome component TSG101 interacts with SPHK1 for encapsulation into EVs. We also demonstrated the release of an enzymatically active form of SPHK1 modulates the level of S1P by the maturation of sphingosine and converting it into S1P. We then showed that S1P causes immunosuppressive effects by inhibiting the actions of T cells through PD‐L1 upregulation in tumor cells. Aberrant S1P signaling has been reported in ovarian cancer^[^ ^21^ ^]^ and our data show that S1P plays a significant role in tumor promotion by upregulating PD‐L1 on tumor cells to inhibit T cell activity.

Recently, the role of tumor‐associated SPHK1 in immunosuppression was reported only in melanoma;^[^ ^22^ ^]^ where Imbert et al. showed that silencing SPHK1 enhanced the immune response, reduced the accumulation of Tregs in tumors, and increased the efficacy of anti‐PD‐1 therapy in melanoma. Our qPCR array of all immune‐checkpoint genes in conjunction with TCGA data showed that SPHK1 or S1P directly associates with several immune checkpoints in ovarian cancer cells. Using genetically modified SPHK1^−/−^ mice, we found an enhanced antitumor immune response of CD8 and CD4 T cells. Further blocking SPHK1 with its inhibitor, PF543, also improved the cytotoxicity of those T cells in tumor ascites. Collectively, such data support a role for SPHK1 in immunosuppression in different types of cancer, making it an important therapeutic target.^[^ ^22^, ^23^ ^]^

Upregulation of PD‐L1 on tumor cells helped to develop well‐known checkpoint inhibitor therapies for cancer and it is widely accepted that PD‐L1 expression in tumor cells is controlled by either interferon receptor signaling^[^ ^24^ ^]^ or IL6/JAK/STAT3 signaling axes.^[^ ^25^ ^]^Here, we found another signaling axis that regulates PD‐L1 expression on tumor cells: S1P upregulates PD‐L1 expression in ovarian cancer cells via the E2F1 transcription factor. Although E2F1 is the key regulator, our transcription factor profiling uncovered several other transcription factors that are both upregulated by S1P stimulation and required for S1P‐mediated PD‐L1 upregulation. We confirmed that E2F1 directly binds onto the PD‐L1 promoter to enhance its transcription by chromatin immunoprecipitation.

S1P is known to regulate T cell egress and trafficking from the lymphoid organ into the blood and S1PR1's role in this phenomenon is well established. In a recent study, Chakraborty et al. showed that blocking intracellular SPHK1/S1P signaling improved the antitumor immunity of T cells.^[^ ^2^ ^]^ They also determined that PPAR*γ* is involved in this outcome, which increases lipolysis‐dependent energy generation and prolongs the antitumor response. Here, we showed that, in the presence of S1P, CD8+ T cells became less cytotoxic and increased their expression of exhaustion markers. We also found that SPHK1‐packaged EVs produced similar effects, confirming our hypothesis that SPHK1‐EVs increase S1P levels in the tumor microenvironment. This increase exerts downstream immunosuppressive effects. S1P‐treated CD8+ T cells also secreted lower levels of cytotoxic cytokines such as IL‐17, TNF alpha, and IFN*γ*, which further suggest that S1P is immunosuppressive in the tumor microenvironment.

In summary, we show for the first time that S1P signaling affects T cell cytotoxicity and regulates PD‐L1 expression by tumor cells. Blocking SPHK1 with PF543 in vivo increased T cell cytotoxicity and reduced PD‐L1 expression on cancer cells. Furthermore, combining anti‐PD‐1 antibody with PF543 increased mouse survival and decreased tumor burden, suggesting that a combination of an S1P/SPHK1 blocker with anti‐PD‐1 therapy may improve the efficacy of immune checkpoint blockers in human ovarian cancer. Overall, our data uncovered a previously unrecognized oncogenic mechanism of how SPHK1‐packaged EVs upregulate S1P in the ovarian cancer microenvironment, enhancing immune suppression through T cell exhaustion.

## 4 Experimental Section

### Patient Samples

Normal and ovarian cancer samples were collected with informed consent from patients under a protocol approved by the Institutional Review Board of Medical College of Wisconsin in accordance with the recognized ethical guidelines of the Declaration of Helsinki. All samples were obtained from the Cancer Center and Department of Obstetrics and Gynecology, Froedtert Hospital, Medical College of Wisconsin.

### Cell Lines

OVCAR5, NIH‐OVACR3, and OVCAR4 cells were purchased from the National Cancer Institute (NCI) cell line repository. HeyA8 cells were received from the Characterized Cell Line Core Facility at MD Anderson Cancer Center, Houston, TX, USA. All cancer cell lines were cultured in DMEM medium (Sigma‐Aldrich, Saint Louis, MO) supplemented with 10% fetal bovine serum (FBS, Atlanta Biologicals, GA, USA), penicillin (100 U mL^−1^), and streptomycin (0.1 mg mL^−1^) (Sigma‐Aldrich). ID8 murine ovarian cancer cells were purchased from Sigma‐Aldrich. ID8 Trp53^−/−^;Brca2^−/−^ was gifted by Dr Iain A. McNeish (Queen Mary University of London). ID8 cells were cultured in DMEM with 4% FBS, Pen‐strep, and 1x ITS solution. Cells were routinely tested and deemed free of mycoplasma, using the MycoSensor PCR assay kit (Agilent,). IDEXX Bioanalytics (Columbia, MO) characterized short tandem repeat (STR) profiling to confirm the cell lines authenticity.

### Tumor Models and Treatment

Female athymic nude mice (CrTac: NCr‐Foxn1nu, Taconic Laboratories, RRID: IMSR_TAC:ncrnu) 4–6 weeks old were maintained under specific pathogen‐free conditions in accordance with guidelines and therapeutic interventions approved by the Institutional Animal Care and Use Committee at the Medical College of Wisconsin. For the tumorgenicity study, HeyA8 cells that ectopically expressed SPHK1, and control cells were trypsinized, washed, and resuspended in Hanks' balanced salt solution (Gibco, Carlsbad, CA). Then 0.25 × 10^6^ cells per mouse were orthotopically injected into the ovarian bursa of anesthetized female nude mice (*n* = 10). C57BL/6 mice 4–6‐weeks old were purchased from the Jackson Laboratory. For all black‐mouse experiments, 5 × 10^6^ ID8 or ID8 Trp53^−/−^; Brca2^−/−^ cells were orthotopically injected into the right ovary. After 10 d of tumor establishment, the tumor‐bearing mice received PF543 (20 mg per mouse, 3x/week; *i.p*.). In combination therapy, anti‐PD1 Ab (200 µg mouse^−1^; 3x/week; *i.p*.) was injected along with pF543. Mice were monitored for tumor growth once every week by bioluminescence imaging using the Xenogen IVIS100 imaging system. The rest of the protocols are given as methods in the Supporting Information.

## Conflict of Interest

The authors declare no conflict of interest.

## Author Contributions

P.G., P.C.‐R., and S.P. designed the study and wrote the paper, and all authors revised the paper for intellectual content and approved the final version. P.G., I.P.K., S.M., J.G., A.G., D.P., P.T., and S.K. executed the experiments and analyzed the data. S.‐W.T. analyzed the RNA sequencing data from TCGA. B.F.V., D.K.V., and K.Y.J.Z. performed the computational biology work for interaction. P.G. and S.P. wrote and revised the paper. L.M., B.F.V., W.C., K.Y.J.Z., D.D.V., P.C.‐R., and S.P. reviewed the final paper.

## Supporting information

Supporting Information

## Data Availability

The data that support the findings of this study are available in the Supporting Information of this article.
