# Supplementary material for: Tumor Derived Extracellular Vesicles Drive T Cell Exhaustion in Tumor Microenvironment through Sphingosine Mediated Signaling and Impacting Immunotherapy Outcomes in Ovarian Cancer
Source: Adv Sci (Weinh). 2022 Mar 15;9(14):2104452. doi: 10.1002/advs.202104452 (PMC9108620; doi:10.1002/advs.202104452)
Supplement: Supplementary file 1 — Supporting Information [file ADVS-9-2104452-s001.pdf]

Tumor Derived Extracellular Vesicles Drive T Cell Exhaustion in Tumor Microenvironment through Sphingosine Mediated Signaling and Impacting Immunotherapy Outcomes in Ovarian Cancer

Prachi Gupta[1], Ishaque Pulikkal Kadamberi[1], Sonam Mittal[1], Shirng-Wern Tsaih[1], Jasmine George[1], Sudhir Kumar[1], Dileep K. Vijayan[2,5], Anjali Geethadevi[1], Deepak Parashar[1], Paytsar Topchyan[3], Lindsey McAlarnen[1], Brian F Volkman[4], Weiguo Cui[3], Kam Y. J. Zhang[5], Dolores Di Vizio[6], Pradeep Chaluvally-Raghavan[1,7,8], Sunila Pradeep[1,7,8]

Supplementary Figures

Supplementary Tables

Supplementary Methods

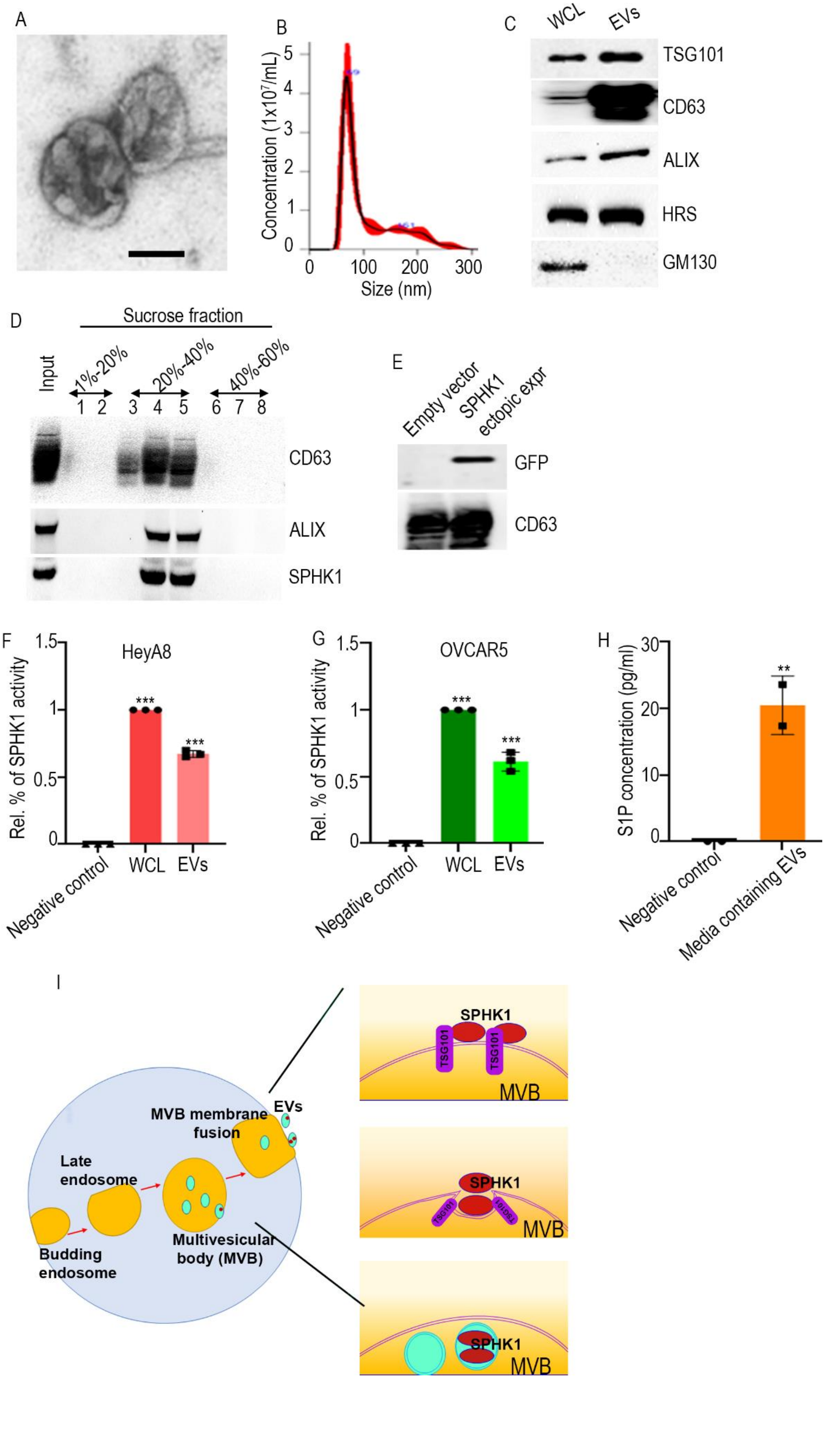

A
B
Concentration (1x10^7/mL)
Size (nm)
C
WCL
EVs
TSG101
CD63
ALIX
HRS
GM130
D
Sucrose fraction
Input
1%-20%
20%-40%
40%-60%
1 2 3 4 5 6 7 8
CD63
ALIX
SPHK1
E
Empty vector
SPHK1 ectopic expr
GFP
CD63
F
HeyA8
Rel. % of SPHK1 activity
Negative control
WCL
EVs
G
OVCAR5
Rel. % of SPHK1 activity
Negative control
WCL
EVs
H
S1P concentration (pg/ml)
Negative control
Media containing EVs
I
SPHK1
TSG101
MVB
EVs
MVB membrane fusion
Late endosome
Multivesicular body (MVB)
Budding endosome

Sup Figure 1. A) Representative TEM image of purified EVs from HeyA8 cells. Scale bar, 50nm. B) Characterization of purified EVs using nanoparticle tracking (NTA). C) Western blotting for EV positive markers (TSG101, CD63, ALIX, HRS) and negative marker GM130. D) Western blotting for SPHK1 and the EV markers CD63 and Alix in different sucrose gradient fractions. E) GFP-tagged SPHK1 was ectopically expressed in HeyA8 cells, and its presence in EVs was observed by western blotting. Relative SPHK1 activity was measured by an ELISA based method in whole cell lysate and EVs from HeyA8 (F) and OVCAR5 (G) cells. All results are means of three technical replicates ± SEM. ***$p<0.001$ compared to negative control by one-way ANOVA. H) S1P concentration was measured by mass spectrometry in culture media incubated with EVs. Media without EVs severed as negative control. Results are mean of two technical replicates ± SEM. **$p<0.01$ compared to negative control by student's *t*-test. I) Schema showing the interaction of TSG101 with SPHK1 during EVs biogenesis that leads to SPHK1 secretion into EVs.

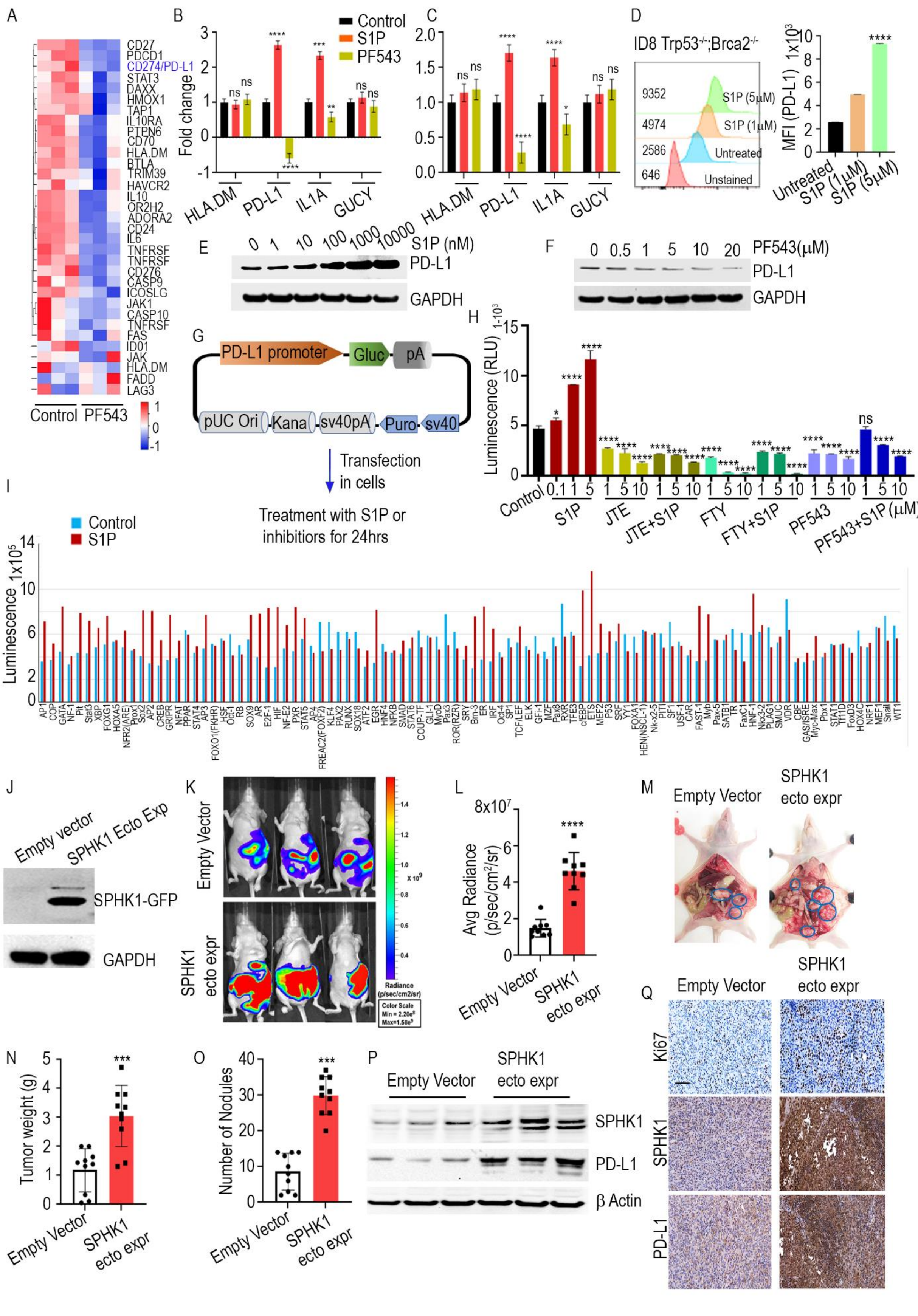
A
CD27
PDCD1
CD274/PD-L1
STAT3
DAXX
HMOX1
TAP1
IL10RA
PTPN6
CD70
HLA.DM
BTLA
TRIM39
HAVCR2
IL10
OR2H2
ADORA2
CD24
IL6
TNFRSF
TNFRSF
CD276
CASP9
ICOSLG
JAK1
CASP10
TNFRSF
FAS
ID01
JAK
HLA.DM
FADD
LAG3
Control
PF543
1
0
-1
B
Control
S1P
PF543
Fold change
HLA.DM
PD-L1
IL1A
GUCY
C
HLA.DM
PD-L1
IL1A
GUCY
D
ID8 Trp53-/-;Brca2-/-
9352
S1P (5μM)
4974
S1P (1μM)
2586
Untreated
646
Unstained
MFI (PD-L1) 1x10³
Untreated
S1P (1μM)
S1P (5μM)
E
0
1
10
100
1000
10000
S1P (nM)
PD-L1
GAPDH
F
0
0.5
1
5
10
20
PF543(μM)
PD-L1
GAPDH
G
PD-L1 promoter
Gluc
pA
pUC Ori
Kana
sv40pA
Puro
sv40
Transfection
in cells
Treatment with S1P or
inhibitions for 24hrs
H
Luminescence (RLU)
Control
S1P
JTE
JTE+S1P
FTY
FTY+S1P
PF543
PF543+S1P (μM)
I
Control
S1P
Luminescence 1x10⁵
J
Empty vector
SPHK1 Ecto Exp
SPHK1-GFP
GAPDH
K
Empty Vector
SPHK1
ecto expr
Radiance
(p/sec/cm2/sr)
Color Scale
Min = 2.20e8
Max=1.58e9
L
Avg Radiance
(p/sec/cm²/sr)
8x10⁷
Empty Vector
SPHK1
ecto expr
M
Empty Vector
SPHK1
ecto expr
N
Tumor weight (g)
Empty Vector
SPHK1
ecto expr
O
Number of Nodules
Empty Vector
SPHK1
ecto expr
P
Empty Vector
SPHK1
ecto expr
SPHK1
PD-L1
β Actin
Q
Empty Vector
SPHK1
ecto expr
Ki67
SPHK1
PD-L1

Sup Figure 2. A) A human immune checkpoint qPCR array was performed using cDNA prepared from OVCAR5 cells treated with the S1P signaling inhibitor PF543 (10 μM). Gene expression is represented as Log2 of Ct values; $p<0.05$ compared to untreated control. Only genes that were ≥1.5-fold downregulated (blue) or upregulated (red) were included in the heat map. Ovarian cancer cell lines B) HeyA8 and C) OVCAR4 were treated with S1P (100 nM) or PF543 (10 μM) for 24 h, and a qPCR array was performed to identify the top four genes upregulated by S1P. Mean ± SEM fold-change values of the indicated genes were shown with respect to control. **$p<0.01$ ***$p<0.001$ ****$p<0.0001$ (one-way ANOVA). The ovarian cancer cell line ID8 $Trp53^{-/-}$;$Brca2^{-/-}$ was stimulated with the indicated doses of S1P for 24 h, and expression of PD-L1 was detected by D) flow cytometry, Mean fluorescent intensity (MFI) were calculated by Flowjo for triplicate experiments. Data is presented as mean ± SEM. ****$p<0.0001$ (one-way ANOVA) and E) western blotting. F) ID8 $Trp53^{-/-}$;$Brca2^{-/-}$ cells were treated with PF543 for 24 h at the indicated doses, and changes in PD-L1 expression were detected by western blotting. G) Schematic diagram of a PD-L1-promotor luciferase reporter assay performed with S1P (10 μM), PF543 (10 μM), FTY720 (10 μM), and JTE013 (10 μM) alone or in combination for 24 h after transfection with vector. H) Luciferase activity was measured in culture medium after OVACR5 cells were treated with the indicated drugs. **$p<0.01$ ***$p<0.001$ ****$p<0.0001$ (one-way ANOVA). I) Bar graph gives the results of a transcription factor array assay performed with OVCAR5 cells stimulated with S1P (100 nM) for 6 h. The x-axis shows the transcription factors, and the y-axis shows the luminescence reading at the end of the assay. J) GFP-tagged SPHK1 was ectopically expressed in HeyA8 cells, and its presence in whole-cell lysates was observed by western blotting. K) Ovarian cancer xenograft mouse models using 1.2×10^6 luciferase-tagged HeyA8 cells were orthotopically injected into the right ovary bursa of female athymic nude mice (N=9 mice/group). Controls were empty vector and SPHK1-expressing vector. The mice from both groups were imaged using biophotonic

IVIS, and representative photographs were presented. (L) Bar graph indicates the average radiance of luminescence intensity from mice (N=9) in each group. Result is shown as mean with 95% CI. ****p<0.0001 (Student's *t*-test). (M) Representative image of a nude mouse from the control and SPHK1-overexpression groups that were surgically opened. Areas circled in blue indicate tumor growth and metastatic locations. (N) Primary and disseminated tumors were collected from M, and total tumor weight was recorded and mean± SEM was plotted. ***p<0.001(Student's *t*-test). (O) Bar graph shows the mean of number of tumor nodules in each group. ***p<0.001(Student's *t*-test). (P) Western blot analysis of lysates collected from tumor tissues selected from each group (N=3), using the indicated antibodies. β-actin was used as a loading control. (Q) Immunohistochemical (IHC) analysis of tumor tissues selected from each group (N=3) was performed for the indicated antibodies. Positivity is shown as brown staining of DAB. All results are means of three technical replicates ± SEM. **p<0.01 ***p<0.001 ****p<0.0001 compared to control by Student's *t*-test otherwise indicated.

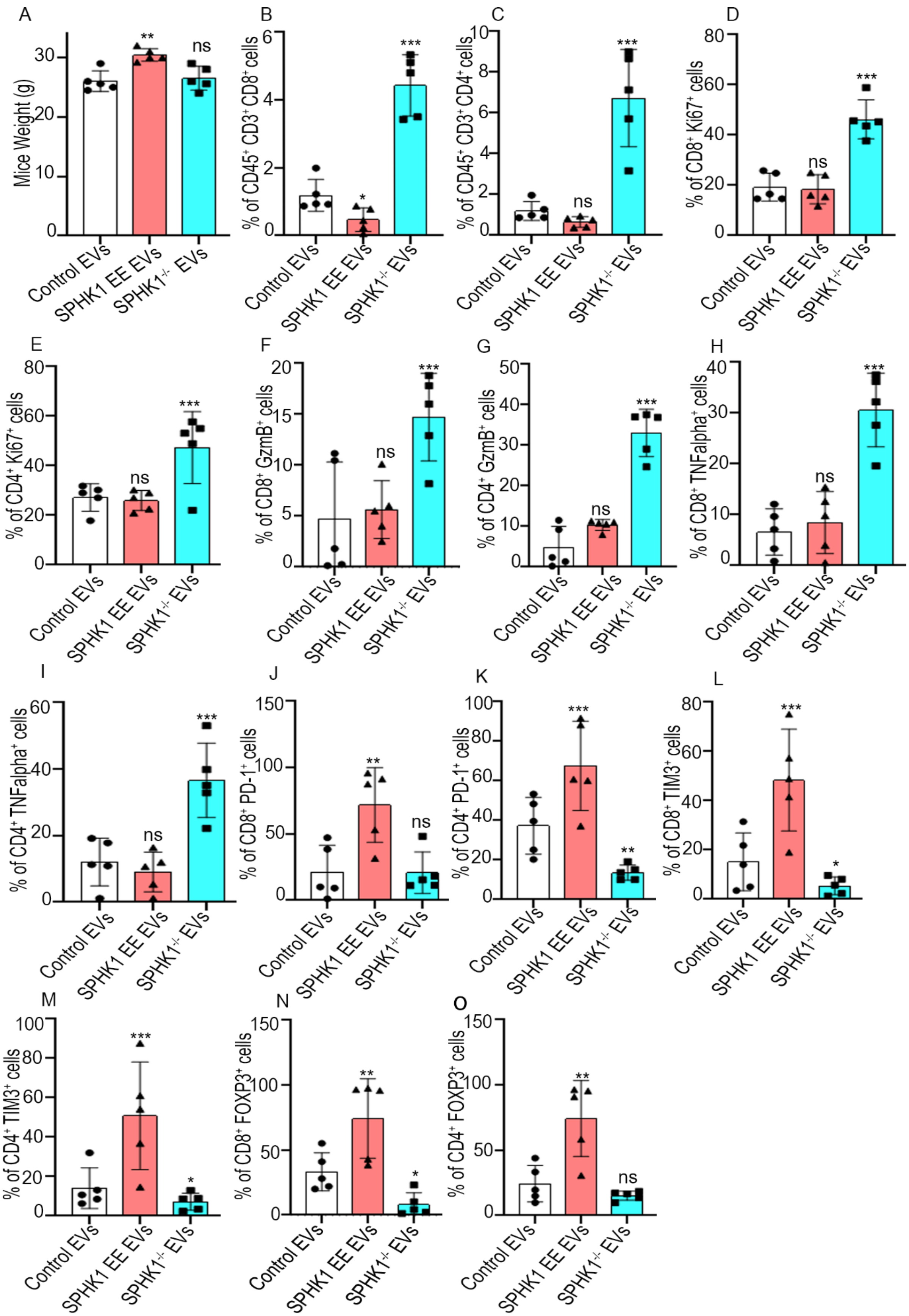
A
Mice Weight (g)
0
10
20
30
**
ns
B
% of CD45+ CD3+ CD8+ cells
0
2
4
6
*
***
C
% of CD45+ CD3+ CD4+ cells
0
2
4
6
8
10
ns
***
D
% of CD8+ Ki67+ cells
0
20
40
60
80
ns
***
E
% of CD4+ Ki67+ cells
0
20
40
60
80
ns
***
F
% of CD8+ GzmB+ cells
0
5
10
15
20
ns
***
G
% of CD4+ GzmB+ cells
0
10
20
30
40
50
ns
***
H
% of CD8+ TNFalpha+ cells
0
10
20
30
40
ns
***
I
% of CD4+ TNFalpha+ cells
0
20
40
60
ns
***
J
% of CD8+ PD-1+ cells
0
50
100
150
**
ns
K
% of CD4+ PD-1+ cells
0
20
40
60
80
100
***
**
L
% of CD8+ TIM3+ cells
0
20
40
60
80
***
*
M
% of CD4+ TIM3+ cells
0
20
40
60
80
100
***
*
N
% of CD8+ FOXP3+ cells
0
50
100
150
**
*
O
% of CD4+ FOXP3+ cells
0
50
100
150
**
ns
Control EVs
SPHK1 EE EVs
SPHK1-/- EVs

Sup Figure 3. A) Bar graph indicates average mouse weight with the indicated EVs. ***p*<0.01 compared to control by one-way ANOVA. Implantation of RAB27a$^{-/-}$ ID8 cells into the ovary of C57BL/6 mice and subsequent treatment with the indicated EVs (N=5 mice/group). Flow cytometric quantification of the percentages of B) CD8+ and C) CD4+ cells among CD45+ and CD3+ cells in ascites after the mice were injected with indicated EVs. Quantification of Ki67+ cells among (D) CD8+ and E) CD4+ T cells in ascites (N=5/group). Quantification of Granzyme B+ cells among F) CD8+ cells and G) CD4+ T cells in ascites (N=5/group). Quantification of TNF alpha+ cells among H) CD8+ cells and I) CD4+ T cells in ascites (N=5/group). Quantification of PD-1+ cells among J) CD8+ cells and K) CD4+ T cells in ascites (N=5/group). Quantification of TIM3+ cells among L) CD8+ cells and M) CD4+ T cells in ascites (N=5/group). Percentages of FOXP3+ cells, N) CD8+ cells, and O) CD4+ T cells among CD45+ and CD3+ cells in ascites of mice (N=5/group). All results are shown as means ± SEM **p*<0.05 ***p*<0.01 ***p<0.001 compared to control EVs by one-way ANOVA.

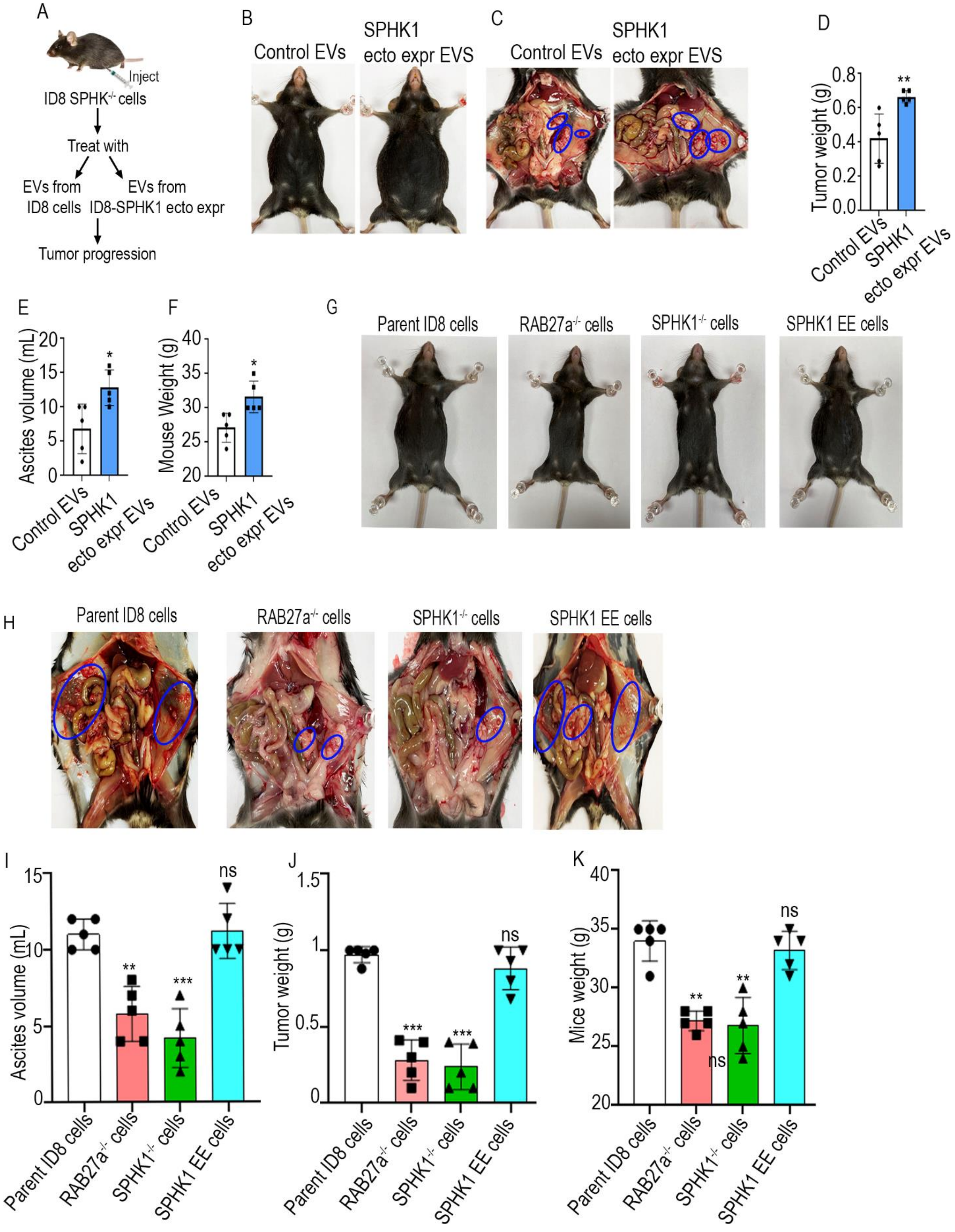

A
Inject
ID8 SPHK-/- cells
Treat with
EVs from ID8 cells
EVs from ID8-SPHK1 ecto expr
Tumor progression
B
Control EVs
SPHK1 ecto expr EVS
C
Control EVs
SPHK1 ecto expr EVS
D
Tumor weight (g)
0.0
0.2
0.4
0.6
0.8
**
Control EVs
SPHK1 ecto expr EVs
E
Ascites volume (mL)
0
5
10
15
20
*
Control EVs
SPHK1 ecto expr EVs
F
Mouse Weight (g)
20
25
30
35
40
*
Control EVs
SPHK1 ecto expr EVs
G
Parent ID8 cells
RAB27a-/- cells
SPHK1-/- cells
SPHK1 EE cells
H
Parent ID8 cells
RAB27a-/- cells
SPHK1-/- cells
SPHK1 EE cells
I
Ascites volume (mL)
0
5
10
15
**
***
ns
Parent ID8 cells
RAB27a-/- cells
SPHK1-/- cells
SPHK1 EE cells
J
Tumor weight (g)
0
0.5
1
1.5
***
***
ns
Parent ID8 cells
RAB27a-/- cells
SPHK1-/- cells
SPHK1 EE cells
K
Mice weight (g)
20
25
30
35
40
**
**
ns
ns
Parent ID8 cells
RAB27a-/- cells
SPHK1-/- cells
SPHK1 EE cells

Sup Figure 4. A) Implantation of SPHK1$^{-/-}$ ID8 cells into the ovary of C57BL/6 mice treated with the indicated EVs (N=5 mice/group). B) By the end of treatment, the mice treated with ectopically expressed EVs containing SPHK1 had a high volume of ascites, whereas the mice treated with control EVs had a smaller volume of ascites. C) Peritoneal cavity of mice showing tumor locations (blue circles) in all treatment groups. Bar graphs indicate mean D) tumor weight, E) ascites volume, and F) body weight with SEM for the mice treated with the EVs. *p<0.05 **p<0.01 compared to control by Student's *t*-test. G) Implementation of parental ID8 cells, RAB27a$^{-/-}$ ID8 cells, SPHK1$^{-/-}$ ID8 cells and SPHK1 ectopic expressing (EE) ID8 cells (N=5 mice/group) into the ovary of C57BL/6 mice. General appearance of mice at the end of treatment. Mice implanted with SPHK1 EE cells showed higher volume of ascites compared to RAB27a$^{-/-}$ ID8 cells and SPHK1$^{-/-}$ ID8 cells group. H) Peritoneal cavity of mice showing tumor locations (blue circles) in all groups. Bar graphs indicate mean I) ascites volume, J) tumor weight, and K) body weight with SEM for the mice injected with indicated ID8 cells. **p<0.01 ***p<0.001 compared to parental ID8 cells by one-way ANOVA.

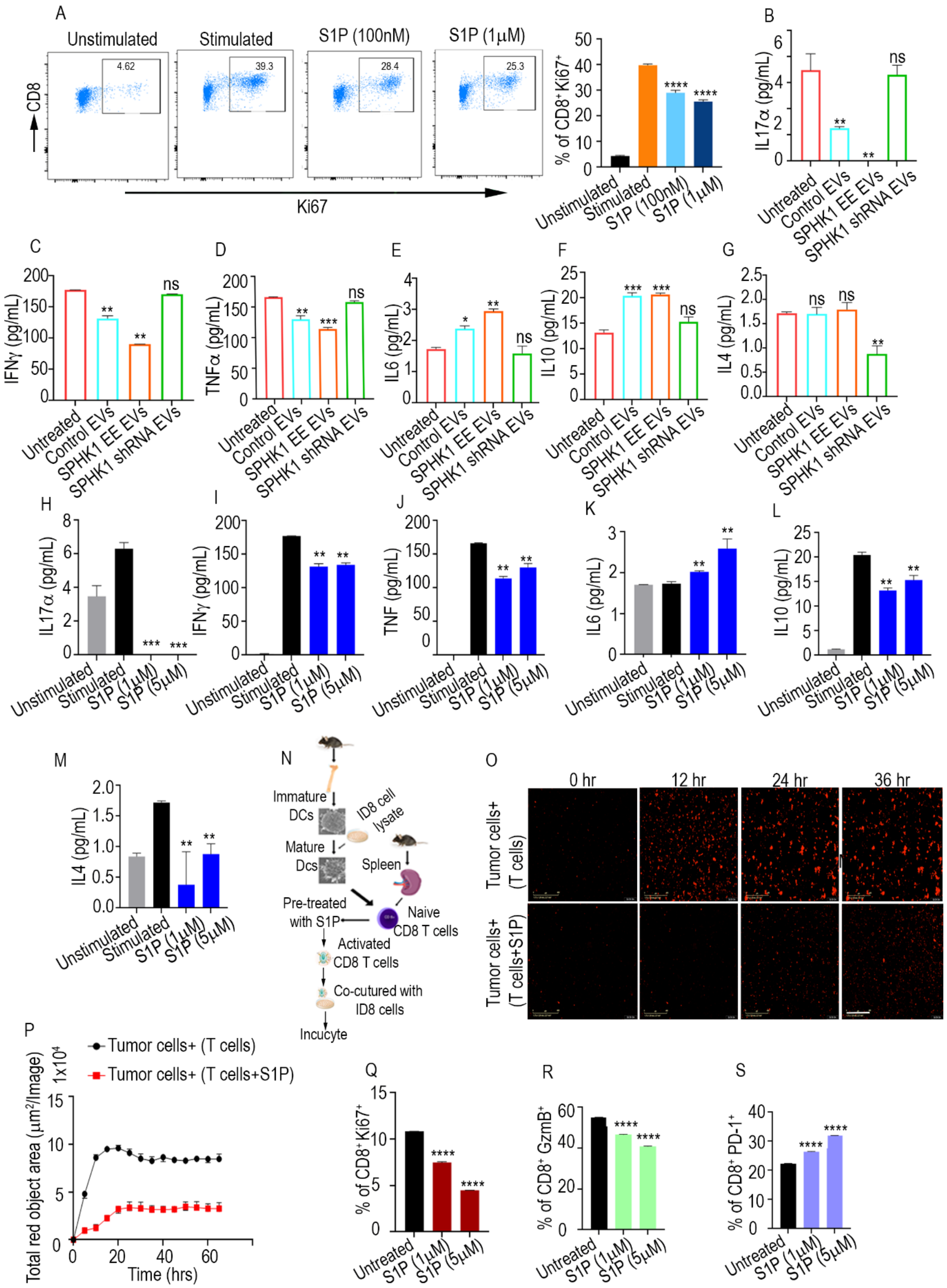

Sup Figure 5. A) Human blood CD8+ T cells were stimulated with S1P, and Ki67 was measured by flow cytometry. Percentages of CD8+ Ki67+ cells were determined by FlowJo. Adjacent bar diagrams represent means ± SEM of cumulative data from three experiments. ****p<0.0001 (one-way ANOVA). (B-G) A human cytokine bead array was performed to determine levels of the indicated cytokines in indicated EVs treated human CD8+ T cells. Results are presented as means ± SEM of technical triplicates. *p<0.05 **p<0.01 ***p<0.001 compared to stimulated and untreated T cells by one-way ANOVA. (H-M) A human cytokine bead array was performed to determine levels of the indicated cytokines in S1P-treated human CD8+ T cells. Results are presented as means ± SEM of technical triplicates. **p<0.01 ***p<0.001 compared to stimulated and untreated T cells by one-way ANOVA. N) Schema is showing isolation of dendritic cells and CD8 T cells from mice and activation of T cells in the presence of S1P in preparation for an IncuCyte and flow cytometry experiment. O) An IncuCyte experiment was performed with activated T cells with or without S1P and ID8 tumor cells. Red color shows staining by annexin-v dye, indicating cell death. P) The total red object area is the IncuCyte count indicating cell death at different time points. Median and error was plotted with 95% CI. 2way ANOVA is applied to know the significance. Q-S) Mouse CD8+ T cells were isolated from spleen and stimulated with S1P. Staining of Ki67, granzyme B, and PD-1 was measured by flow cytometry. Percentages of CD8+ KI67+ cells, CD8+ granzyme B+ cells, and CD8+ PD-1+ cells were determined by FlowJo. Results are means of three technical replicates ± SEM. ****p<0.0001 compared to control by one-way ANOVA.

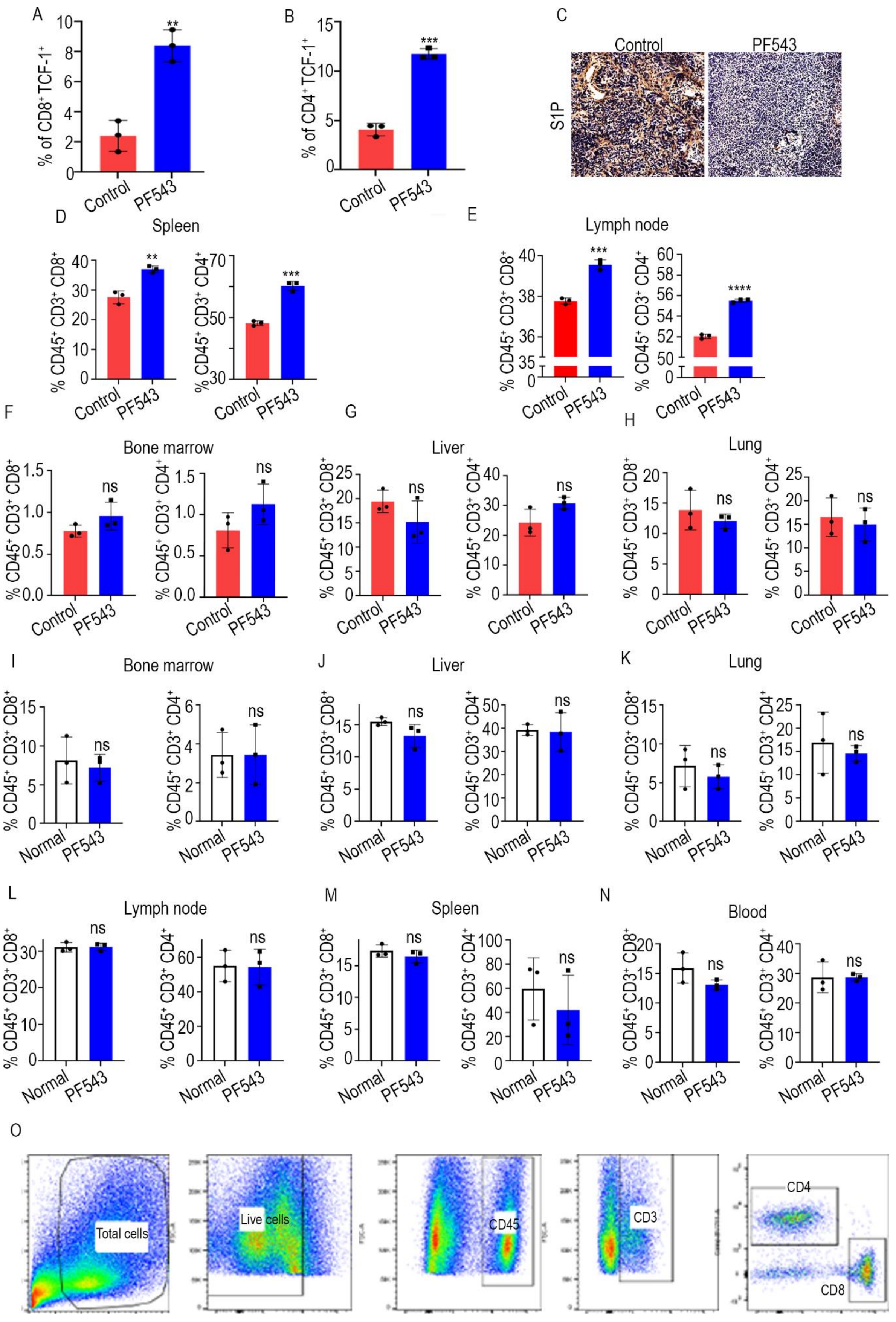
A
% of CD8⁺TCF-1⁺
**
Control
PF543
B
% of CD4⁺TCF-1⁺
***
Control
PF543
C
Control
PF543
S1P
D
Spleen
% CD45⁺ CD3⁺ CD8⁺
% CD45⁺ CD3⁺ CD4⁺
**
***
Control
PF543
E
Lymph node
% CD45⁺ CD3⁺ CD8⁺
% CD45⁺ CD3⁺ CD4⁺
***
****
Control
PF543
F
Bone marrow
G
Liver
H
Lung
% CD45⁺ CD3⁺ CD8⁺
% CD45⁺ CD3⁺ CD4⁺
ns
Control
PF543
I
Bone marrow
J
Liver
K
Lung
% CD45⁺ CD3⁺ CD8⁺
% CD45⁺ CD3⁺ CD4⁺
ns
Normal
PF543
L
Lymph node
M
Spleen
N
Blood
% CD45⁺ CD3⁺ CD8⁺
% CD45⁺ CD3⁺ CD4⁺
ns
Normal
PF543
O
Total cells
Live cells
CD45
CD3
CD4
CD8

Sup Figure 6. A-B) Flow cytometric quantification of the percentages of TCF1+ CD8+ and CD4+ cells in ascites from control vs PF543 treated group. (N=3) C) IHC staining of S1P in tumor tissues from control and PF543 treatment group. D-H) Flow cytometric quantification of the percentages of CD8+ and CD4+ cells among CD45+ and CD3+ cells in the (D) spleen, (E) lymph nodes, (F) bone marrow, (G) liver, and (H) lung in control vs. PF543- treated mice bearing ID8 tumors (N=3/group). I-N) Flow cytometric quantification of the percentages of CD8+ and CD4+ cells among CD45+ and CD3+ cells in the (I) bone marrow, (J) liver (K) lung, (L) lymph nodes, (M) spleen, and (N) blood in control and PF543-treated normal mice (N=3/group). O) Representative contour plots showing the general gating strategy used to identify CD8+ and CD4+ T cells in the mouse samples. Adjacent bar diagrams represent cumulative data from three experiments. Results are presented as means ± SEM. **p<0.01 ***p<0.001 ****p<0.0001 in comparison to control or normal (Student's t test).

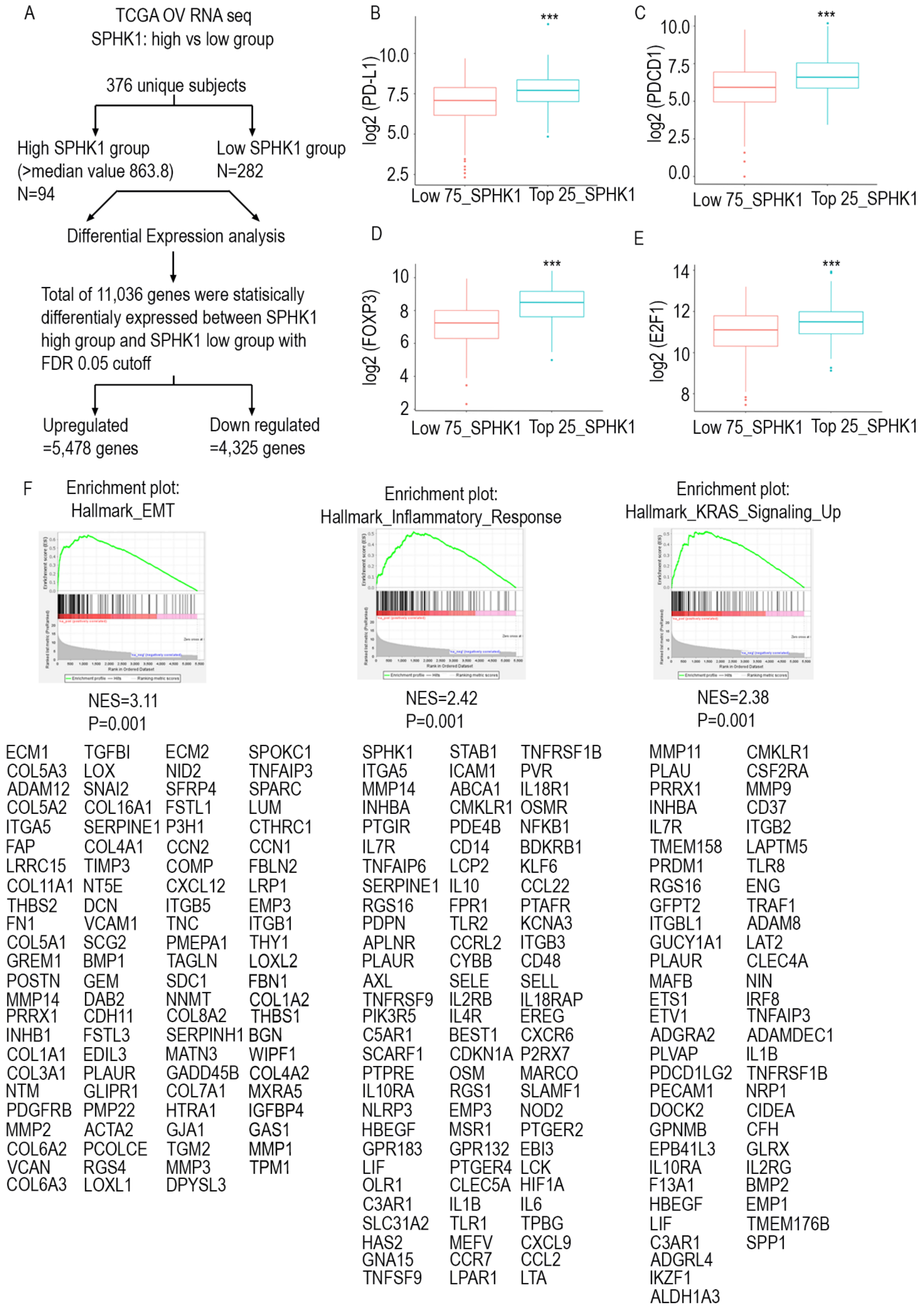
A
TCGA OV RNA seq
SPHK1: high vs low group
376 unique subjects
High SPHK1 group
(>median value 863.8)
N=94
Low SPHK1 group
N=282
Differential Expression analysis
Total of 11,036 genes were statisically differentialy expressed between SPHK1 high group and SPHK1 low group with FDR 0.05 cutoff
Upregulated =5,478 genes
Down regulated =4,325 genes
B
log2 (PD-L1)
Low 75_SPHK1
Top 25_SPHK1
***
C
log2 (PDCD1)
Low 75_SPHK1
Top 25_SPHK1
***
D
log2 (FOXP3)
Low 75_SPHK1
Top 25_SPHK1
***
E
log2 (E2F1)
Low 75_SPHK1
Top 25_SPHK1
***
F
Enrichment plot: Hallmark_EMT
NES=3.11
P=0.001
Enrichment plot: Hallmark_Inflammatory_Response
NES=2.42
P=0.001
Enrichment plot: Hallmark_KRAS_Signaling_Up
NES=2.38
P=0.001
ECM1 TGFBI ECM2 SPOKC1
COL5A3 LOX NID2 TNFAIP3
ADAM12 SNAI2 SFRP4 SPARC
COL5A2 COL16A1 FSTL1 LUM
ITGA5 SERPINE1 P3H1 CTHRC1
FAP COL4A1 CCN2 CCN1
LRRC15 TIMP3 COMP FBLN2
COL11A1 NT5E CXCL12 LRP1
THBS2 DCN ITGB5 EMP3
FN1 VCAM1 TNC ITGB1
COL5A1 SCG2 PMEPA1 THY1
GREM1 BMP1 TAGLN LOXL2
POSTN GEM SDC1 FBN1
MMP14 DAB2 NNMT COL1A2
PRRX1 CDH11 COL8A2 THBS1
INHB1 FSTL3 SERPINH1 BGN
COL1A1 EDIL3 MATN3 WIPF1
COL3A1 PLAUR GADD45B COL4A2
NTM GLIPR1 COL7A1 MXRA5
PDGFRB PMP22 HTRA1 IGFBP4
MMP2 ACTA2 GJA1 GAS1
COL6A2 PCOLCE TGM2 MMP1
VCAN RGS4 MMP3 TPM1
COL6A3 LOXL1 DPYSL3
SPHK1 STAB1 TNFRSF1B
ITGA5 ICAM1 PVR
MMP14 ABCA1 IL18R1
INHBA CMKLR1 OSMR
PTGIR PDE4B NFKB1
IL7R CD14 BDKRB1
TNFAIP6 LCP2 KLF6
SERPINE1 IL10 CCL22
RGS16 FPR1 PTAFR
PDPN TLR2 KCNA3
APLNR CCRL2 ITGB3
PLAUR CYBB CD48
AXL SELE SELL
TNFRSF9 IL2RB IL18RAP
PIK3R5 IL4R EREG
C5AR1 BEST1 CXCR6
SCARF1 CDKN1A P2RX7
PTPRE OSM MARCO
IL10RA RGS1 SLAMF1
NLRP3 EMP3 NOD2
HBEGF MSR1 PTGER2
GPR183 GPR132 EBI3
LIF PTGER4 LCK
OLR1 CLEC5A HIF1A
C3AR1 IL1B IL6
SLC31A2 TLR1 TPBG
HAS2 MEFV CXCL9
GNA15 CCR7 CCL2
TNFSF9 LPAR1 LTA
MMP11 CMKLR1
PLAU CSF2RA
PRRX1 MMP9
INHBA CD37
IL7R ITGB2
TMEM158 LAPTM5
PRDM1 TLR8
RGS16 ENG
GFPT2 TRAF1
ITGBL1 ADAM8
GUCY1A1 LAT2
PLAUR CLEC4A
MAFB NIN
ETS1 IRF8
ETV1 TNFAIP3
ADGRA2 ADAMDEC1
PLVAP IL1B
PDCD1LG2 TNFRSF1B
PECAM1 NRP1
DOCK2 CIDEA
GPNMB CFH
EPB41L3 GLRX
IL10RA IL2RG
F13A1 BMP2
HBEGF EMP1
LIF TMEM176B
C3AR1 SPP1
ADGRL4
IKZF1
ALDH1A3

Sup Figure. 7. A) Flow chart shows the strategy used to analyze TCGA ovarian cancer RNA seq data for SPHK1 expression. B-E) Box plot analysis, using TCGA dataset, of expression of the indicated genes in the patient groups with low vs. high SPHK1 expression. ***p<0.001 (Student's *t*-test) F) Gene set enrichment analysis of the genes with high SPHK1 expression, using cancer hallmarks database using the Molecular Signatures Database Hallmark Gene Set Collection (GSEA 4.1.0.).

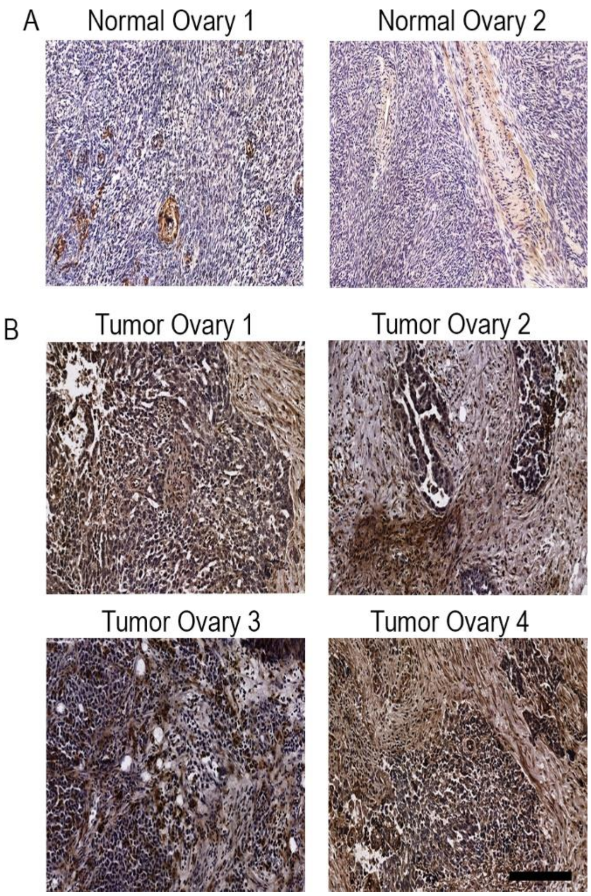

Sup Figure. 8. IHC staining of tissue sections from Human A) Normal ovary and B) Tumor ovary from different subjects. Brown staining is DAB showing positive expression of S1P in tumor microenvironment. 20x images were taken by case viewer. Scale bar=50μM.

Sup Table 1 qPCR array of immune checkpoints and immune cell modulators

Position	Gene Symbol	Fold Change (comparing to control			
		Group 1 (S1P)		Group 2 (PF543)	
		Fold Change	p-Value	Fold Change	p-Value
A01	TAP1	0.67	0.1054	0.49	0.2223
A02	TAP2	0.41	0.1288	1.06	0.8918
A03	JAK	1.15	0.3399	0.64	0.4559
A04	CASP10	0.63	0.2326	0.4	0.1018
A05	CASP3	0.55	0.0974	0.92	0.5772
A06	CASP6	0.63	0.1255	0.27	0.0328
A07	CASP7	0.59	0.0804	0.74	0.1888
A08	JAK1	0.92	0.6318	0.48	0.1359
A09	DAXX	0.81	0.6100	0.23	0.0513
A10	FADD	0.9	0.3937	1.63	0.0079
A11	FAS	0.7	0.5770	0.55	0.2365
A12	HMOX1	0.64	0.3293	0.36	0.1638
B01	STAT3	0.83	0.4385	0.28	0.0718
B02	TNFRSF10A	0.6	0.2173	0.79	0.4233
B03	TNFRSF10B	0.69	0.3311	0.38	0.1857
B04	TNFRSF18	0.76	0.1775	0.13	0.0025
B05	TNFRSF9	0.89	0.6999	0.37	0.0042
B06	TRIM39	0.88	0.2745	0.6	0.0749
B07	GUCY1A3	1.62	0.3049	0.99	0.7716
B08	ADORA2A	0.67	0.0314	0.08	0.0021
B09	ICOSLG	0.58	0.0286	0.24	0.0309
B10	IDO1	0.98	0.7360	0.28	0.0863
B11	OR2H2	0.55	0.0010	0.25	0.0046
B12	IL6	1.48	0.1503	0.21	0.0003
C01	PDCD1	0.65	0.1736	0.43	0.0422

C02	PTPN6	1.04	0.1343	0.5	0.0002
C03	BTLA	0.82	0.7283	0.62	0.0026
C04	CD24	0.95	0.7286	0.17	0.0001
C05	CD27	0.37	0.0358	0.09	0.0020
C06	CD274	2.14	0.0533	0.3	0.0547
C07	CD276	0.17	0.0575	0.37	0.0410
C08	CD70	0.83	0.1734	0.53	0.0006
C09	HAVCR2	0.96	0.8751	0.66	0.1284
C10	HLA-DMA	0.94	0.2514	0.58	0.0016
C11	HLA-DMB	3.21	0.3087	0.42	0.5881
C12	ICOS	1.36	0.1796	1.02	0.8328
D01	IL10	0.74	0.2854	0.64	0.0361
D02	IL10RA	1.49	0.0412	0.56	0.0011
D03	IL10RB	0.85	0.0878	0.73	0.0093
D04	IL18	1.07	0.9068	0.8	0.3451
D05	IL1A	2.92	0.1853	1.36	0.8832
D06	JAK3	0.56	0.0717	0.24	0.0120
D07	MICA	0.8	0.7379	0.07	0.0032
D08	MICB	0.95	0.8508	0.2	0.0120
D09	PDCD1LG2	1.14	0.5236	0.31	0.0023
D10	TNFRSF4	0.47	0.0273	0.39	0.0086
D11	TNFSF14	1.28	0.6939	0.18	0.0528
D12	TNFSF9	0.43	0.0208	0.43	0.2257
E01	CCL2	1.37	0.0007	0.37	0.0000
E02	HRH4	1.3	0.5032	0.57	0.1566
E03	NT5E	0.97	0.6688	0.61	0.0006
E04	PDCD4	1.21	0.0908	0.99	0.8662
E05	CD244	1.15	0.5033	0.97	0.9809
E06	CD28	0.42	0.0515	0.35	0.0293
E07	CD4	0.34	0.0191	0.22	0.0026

E08	CD80	0.45	0.0445	0.46	0.0244
E09	CD86	0.59	0.2063	0.33	0.0728
E10	PIK3CA	1.24	0.0184	0.75	0.0434
E11	PSMB8	0.97	0.6618	0.88	0.0850
E12	PSMB9	0.7	0.2857	0.88	0.5589
F01	PTPN11	1.01	0.9729	0.74	0.0513
F02	LGALS9	0.81	0.2338	0.13	0.0041
F03	VTCN1	0.61	0.2937	0.53	0.0408
F04	IL12B	0.94	0.6828	0.44	0.2421
F05	TUBB	0.68	0.0141	0.78	0.0706
F06	CTLA4	0.52	0.1494	0.44	0.0882
F07	FOXP3	0.53	0.0710	0.35	0.0059
F08	STAT1	1.24	0.0275	1.13	0.1467
F09	FLOT1	1.06	0.2079	0.79	0.0147
F10	TNFRSF 14	0.89	0.1791	0.43	0.0068
F11	LAG3	1.2	0.7175	2.92	0.4969
F12	CD160	0.55	0.0457	0.17	0.0018
G01	CD247	1	0.8961	0.25	0.0692
G02	STAT5A	0.85	0.3886	0.36	0.0424
G03	CD40LG	0.66	0.6016	0.13	0.2048
G04	CD96	1.4	0.4901	0.72	0.4029
G05	IL6ST	1.24	0.7144	0.81	0.3857
G06	IFNG	0.75	0.4155	0.63	0.2111
G07	KIR3DL1	0.41	0.1786	0.18	0.1322
G08	KLRG1	0.78	0.2697	0.24	0.0010
G09	TGFBR2	0.84	0.0696	0.74	0.0240
G10	TNFSF4	1.12	0.4610	0.85	0.0004
G11	FOXO1	1.12	0.4345	0.97	0.8297
G12	IRF1	0.95	0.3523	0.88	0.9515
H01	ACTB	0.65	0.1031	0.72	0.1794

H02	B2M	1.2	0.0353	0.86	0.0982
H03	GAPDH	0.87	0.1051	1.63	0.0074
H04	HPRT1	1.15	0.0477	1	0.9573
H05	RPLP0	1.28	0.0070	0.98	0.8534
H06	GDC	0.98	0.9351	0.82	0.3768

Sup Table 2 E2F1 binding sites predicted by Matinspector on PD-L1 promotor

Matrix ID	Name	Score	Relative score	Start	End	Strand	Predicted sequence
MA0024.2	E2F1	11.7459	0.944735071336	450	460	+	CTCGCGGGAAC
MA0024.2	E2F1	7.51648	0.881105040123	406	416	-	CGCGCGGAAAG
MA0024.1	E2F1	8.83194	0.870250555575	125	132	-	TTTGGCAC
MA0024.1	E2F1	8.83194	0.870250555575	319	326	-	CTTGGCGC
MA0024.1	E2F1	8.32861	0.852930971432	408	415	+	TTCCGCGC
MA0024.2	E2F1	5.62925	0.85271214037	61	71	+	ATGGTGGGAAC
MA0024.3	E2F1	8.68073	0.834170048106	121	132	+	GATGGTGCCAAA
MA0024.2	E2F1	3.31535	0.817900176445	122	132	+	ATGGTGCCAAA
MA0024.2	E2F1	3.29836	0.817644509905	449	459	-	TTCCCGCGAGA
MA0024.3	E2F1	7.17397	0.809082621095	121	132	-	TTTGGCACCATC
MA0024.2	E2F1	2.67957	0.808335054732	371	381	-	GGAGCGCCCAC

Sup Table 3 Ovarian cancer TMA Slide

Female Age	Organ/Anatomic Site	Pathology diagnosis	TNM	Grade	Stage	Type	SPHK 1 staining intensity	PDL1 staing intensity
65	Ovary	Low grade serous carcinoma	T1N0M0	-	I	Malignant	3	3
38	Ovary	Low grade serous carcinoma	T3cN1M0	-	IIIC	Malignant	3	3
51	Ovary	High grade serous carcinoma	T3cN1M0	-	IIIC	Malignant	3	3
22	Ovary	High grade serous carcinoma	T2bN0M0	-	IIB	Malignant	3	3
48	Ovary	Low grade serous carcinoma	T1N0M0	-	I	Malignant	3	3
26	Ovary	High grade serous carcinoma	T3cN1M0	-	IIIC	Malignant	3	3
25	Ovary	Low grade serous carcinoma	T1N0M0	-	I	Malignant	3	3
50	Ovary	High grade serous carcinoma	T2N0M0	*	II	Malignant	2	3
26	Ovary	High grade serous carcinoma	T1cN0M0	-	IC	Malignant	2	3
47	Ovary	High grade serous carcinoma	T1N0M0	-	I	Malignant	3	3
58	Ovary	High grade serous carcinoma	T1N0M0	-	I	Malignant	3	3

57	Ovary	High grade serous carcinoma	T1cN0M0	-	IC	Malignant	3	3
51	Ovary	High grade serous carcinoma	T1aN0M0	*	IA	Malignant	3	3
52	Ovary	High grade serous carcinoma	T2N0M0	-	II	Malignant	3	3
54	Ovary	High grade serous carcinoma	T3cN1M0	-	IIIC	Malignant	3	3
33	Ovary	High grade serous carcinoma	T1N0M0	-	I	Malignant	2	3
56	Ovary	High grade serous carcinoma	T2N0M0	-	II	Malignant	2	3
41	Ovary	High grade serous carcinoma	T1N0M0	-	I	Malignant	2	3
46	Ovary	High grade serous carcinoma	T3aN0M0	-	III	Malignant	3	3
46	Ovary	High grade serous carcinoma	T2cN1M0	*	IIIC	Malignant	2	3
57	Ovary	High grade serous carcinoma	T3cN1M0	-	IIIC	Malignant	2	2
75	Ovary	High grade serous carcinoma	T2N0M0	-	II	Malignant	2	2
54	Ovary	High grade serous carcinoma	T3cN1M0	-	IIIC	Malignant	2	2
49	Ovary	High grade serous carcinoma	T2N0M0	-	II	Malignant	2	2
50	Ovary	High grade serous carcinoma	T1N0M0	-	I	Malignant	3	2
52	Ovary	High grade serous carcinoma	T2N0M0	*	II	Malignant	1	2

47	Ovary	High grade serous carcinoma	T3cN1M0	-	IIIC	Malignant	2	2
34	Ovary	Mucinous adenocarcinoma	T1bN0M0	2	IB	Malignant	2	1
63	Ovary	Mucinous adenocarcinoma	T1aN0M0	1	IA	Malignant	1	1
69	Ovary	Mucinous adenocarcinoma	T1bN0M0	1	IB	Malignant	1	1
46	Ovary	Endometrioid adenocarcinoma	T2N0M0	1--2	II	Malignant	3	2
47	Ovary	Endometrioid adenocarcinoma	T2aN0M0	1--2	IIA	Malignant	1	2
54	Ovary	Endometrioid adenocarcinoma	T1bN0M0	1--2	IB	Malignant	2	2
65	Ovary	Adenocarcinoma (sparse)	T1cN0M0	2	IC	Malignant	2	2
55	Ovary	Endometrioid adenocarcinoma	T1N0M0	2	I	Malignant	3	2
54	Ovary	Endometrioid adenocarcinoma	T1bN0M0	1	IB	Malignant	2	2
43	Ovary	Endometrioid adenocarcinoma	T1cN0M0	2	IC	Malignant		2
55	Ovary	Endometrioid adenocarcinoma with necrosis	T1N0M0	2	I	Malignant	1	2
53	Ovary	Endometrioid adenocarcinoma	T2aN0M0	3	IIA	Malignant	3	2
50	Ovary	Endometrioid adenocarcinoma	T3bN1M0	2	IIIC	Malignant	2	2

51	Ovary	Invasive urothelial carcinoma	T1bN0M0	-	IB	Malignant	2	2
39	Ovary	Invasive urothelial carcinoma	T1aN0M0	-	IA	Malignant	3	2
38	Ovary	Invasive urothelial carcinoma	T1N0M0	-	I	Malignant	3	2
66	Ovary	Invasive urothelial carcinoma with squamous metaplasia	T1aN0M0	-	IA	Malignant	1	2
53	Ovary	Invasive urothelial carcinoma	T1N0M0	-	I	Malignant	1	2
47	Mesentery	Metastatic papillary adenocarcinoma from ovary	-	2	-	Metastasis	3	2
57	Greater omentum	Metastatic papillary adenocarcinoma from ovary	-	2	-	Metastasis	3	2
65	Greater omentum	Metastatic papillary adenocarcinoma from ovary	-	2	-	Metastasis	3	2
59	Mesentery	Metastatic papillary adenocarcinoma from ovary	-	2	-	Metastasis	3	2
28	Greater omentum	Metastatic papillary adenocarcinoma with calcification from ovary	-	2	-	Metastasis	2	2
64	Greater omentum	Metastatic papillary adenocarcinoma from ovary	-	2	-	Metastasis	3	1

50	Greater omentum	Metastatic papillary adenocarcinoma from ovary	-	2	-	Metastasis	3	1
58	Greater omentum	Metastatic adenocarcinoma from ovary	-	3	-	Metastasis	3	1
47	Peritoneum	Metastatic adenocarcinoma from ovary	-	3	-	Metastasis	3	1
49	Peritoneum	Metastatic adenocarcinoma from ovary	-	3	-	Metastasis	1	1
34	Ovary	Borderline serous papillary cystadenoma	-	-	-	Borderline	1	1
34	Ovary	Borderline serous papillary cystadenoma	-	-	-	Borderline	1	1
28	Ovary	Borderline serous papillary cystadenoma	-	-	-	Borderline	1	2
22	Ovary	Borderline mucinous papillary cystadenoma	-	-	-	Borderline	1	2
60	Ovary	Borderline serous papillary cystadenoma	-	-	-	Borderline	1	2
50	Ovary	Borderline serous papillary cystadenoma	-	-	-	Borderline	2	1

37	Ovary	Borderline mucinous papillary cystadenoma	-	-	-	Borderline	1	1
62	Ovary	Serous cystadenoma	-	-	-	Benign	2	2
70	Ovary	Serous cystadenoma	-	-	-	Benign	2	2
49	Ovary	Serous cystadenoma	-	-	-	Benign	2	2
16	Ovary	Serous cystadenoma	-	*	-	Benign	2	2
34	Ovary	Serous cystadenoma	-	-	-	Benign	2	2
22	Ovary	Serous cystadenoma	-	-	-	Benign	2	2
19	Ovary	Mucinous cystadenoma	-	-	-	Benign	2	2
17	Ovary	Mucinous cystadenoma	-	-	-	Benign	2	2
41	Uterus	Mucinous cystadenoma	-	-	-	Benign	2	2
26	Ovary	Mucinous cystadenoma	-	-	-	Benign	2	2
22	Ovary	Mucinous cystadenoma	-	*	-	Benign	2	2
38	Ovary	Mucinous cystadenoma	-	*	-	Benign	2	2
47	Ovary	Mucinous cystadenoma	-	-	-	Benign	2	2

70	Ovary	Mucinous cystadenoma	-	-	-	Benign	2	2
51	Ovary	Mucinous cystadenoma	-	-	-	Benign	2	2
29	Ovary	Mucinous cystadenoma	-	-	-	Benign	2	2
35	Ovary	Mucinous cystadenoma	-	-	-	Benign	2	2
18	Ovary	Mucinous cystadenoma	-	-	-	Benign	2	2
30	Ovary	Adjacent normal ovary tissue	-	-	-	NAT	1	1
39	Ovary	Adjacent normal ovary tissue	-	-	-	NAT	1	1
29	Ovary	Adjacent normal ovary tissue	-	-	-	NAT	1	1
41	Ovary	Adjacent normal ovary tissue	-	-	-	NAT	1	1
62	Ovary	Adjacent normal ovary tissue	-	-	-	NAT	1	1
63	Ovary	Adjacent normal ovary tissue	-	-	-	NAT	1	1
45	Ovary	Adjacent normal ovary tissue	-	-	-	NAT	1	1
48	Ovary	Adjacent normal ovary tissue	-	-	-	NAT	1	1
53	Ovary	Adjacent normal ovary tissue	-	-	-	NAT	1	1
53	Ovary	Adjacent normal ovary tissue	-	-	-	NAT	1	1

57	Ovary	Adjacent normal ovary tissue	-	-	-	NAT	1	1
38	Ovary	Adjacent normal ovary tissue	-	-	-	NAT	1	1
53	Ovary	Adjacent normal ovary tissue	-	-	-	NAT	1	1
59	Ovary	Adjacent normal ovary tissue	-	-	-	NAT	1	1
48	Ovary	Adjacent normal ovary tissue	-	-	-	NAT	1	1
50	Ovary	Adjacent normal ovary tissue	-	-	-	NAT	1	1
52	Ovary	Adjacent normal ovary tissue	-	-	-	NAT	1	1
27	Ovary	Ovary tissue	-	*	-	Normal	1	0
34	Ovary	Ovary tissue	-	*	-	Normal	1	0
19	Ovary	Ovary tissue	-	-	-	Normal	1	0
42	Adrenal gland	Pheochromocytoma (tissue marker)		-		Malignant		

Sup Table 4 List of antibodies used in this study

REAGENT or RESOURCE	SOURCE	IDENTIFIER
Antibodies		
Anti-mouse CD4-BV711	Biolegend	Clone: GK1.5; Cat# 100447; RRID: AB_2564586
Anti-mouse CD8-BV605	Biolegend	Clone: 53-6.7; Cat# 100744; RRID:AB_2562609
Anti-mouse CD45-BV510	Biolegend	Clone: 30-F11; Cat# 103138; RRID:AB_2563061
Anti-mouse CD3-BUV395	BD Horizon	Clone; 145-2C11 (RUO) Cat# 563565; RRID:AB_2738278
Anti-mouse CD3-PE/Cy7	Biolegend	Cat# 100220
Anti-mouse Ki67 Alexa Fluor 647	BD Pharmingen	Clone: N/A; Cat# 561126; RRID:AB_10611874
Anti-mouse Granzyme B eflour 405	Thermo Fisher Scientific	Clone: NGZB; Cat# 48-8898-82; RRID:AB_11149362
Anti-mouse PD1-PE	BD Pharmingen	Clone: N/A; Cat# 561788; RRID:AB_10895570
Anti-mouse TIM3-BV421	BD Biosciences	Clone: 5D12; Cat# 747626; RRID:AB_2744192
Anti-mouse Foxp3-FITC	Thermo Fisher Scientific	Clone: FJK-16s; Cat# 11-5773-82; RRID:AB_465243
Anti-mouse CD45-PE	Thermo Fisher Scientific	Clone: 30-F11; Cat# 12-0451-83; RRID:AB_465669
Anti-mouse CD8-APC efluor 780	Thermo Fisher Scientific	Clone: 53-6.7; Cat# 47-0081-82; RRID:AB_1272185
Anti-mouse CD3-BV510	BD Horizon	Clone: 145-2C11; Cat# 563024; RRID:AB_2737959
PPARg-Alexa Fluor 405	Santa Cruz Biotechnology	Clone: N/A; Cat# SC-7273; RRID:AB_628115
Anti-mouse EpCam-FITC	Thermo Fisher Scientific	Clone: G8.8; Cat# 11-5791-82; RRID:AB_11151709
Anti-mouse PDL1-BV605	Biolegend	Clone: 10F.9G2; Cat# 124321; RRID:AB_2563635
Anti-human CD8-eFlour450	Thermo Fisher Scientific	Clone: RPA-T8; Cat# 48-0088-42: RRID: AB 1272062
Anti-human Ki67-PE-Cy7	BD Pharmingen	Clone: B56; Cat# 561283; RRID: AB 10716060
Anti-human Granzyme B-PE	Thermo Fisher Scientific	Clone: GB11; Cat# GRB04; RRID: AB 2536538

Anti-human PD-1-PE	Thermo Fisher Scientific	Clone: MIH4; Cat# 12-9969-42; AB 10736473
Anti-human Foxp3-PE	Thermo Fisher Scientific	Clone: PCH101; Cat# 12-4776-42; RRID: AB 1518782
SPHK1	Proteintech	Clone: N/A; Cat# 10670-I-AP; RRID:AB_2195809
PDL1(IHC)	Proteintech	Cat# 17952-I-AP
TSG101	Proteintech	Clone: N/A; Cat# 14497-I-AP; RRID:AB_2208090
CD63	Proteintech	Clone: N/A; Cat# 25682-I-AP; RRID:AB_2208090
PDL1	Cell Signaling Technology	Clone: E1L3N®; Cat# 13684S; RRID:AB_2687655
GAPDH	Cell Signaling Technology	Clone: 14C10; Cat# 2118S; RRID:AB_561053
Alix	Santa Cruz Biotechnology	Clone: 1A12; Cat# sc-53540; RRID:AB_673819
Ki67 (IHC)	Cell Signaling Technology	Clone: D3B5; Cat# 12202S; RRID:AB_2620142
Beta-Actin	Cell Signaling Technology	Clone: 13E5; Cat#4970; RRID:AB_2223172
TSG101	Thermo Fisher Scientific	Clone: 4A10; Cat# MA1-23296; RRID:AB_2208088
GFP	Santa Cruz Biotechnology	Clone: NA; Cat# Sc-9996; RRID:AB_627695
Anti-Rabbit HRP	Cell Signaling Technology	Cat#: 7074S: RRID:AB_2099233
Anti-Mouse HRP	Cell Signaling Technology	Cat#: 7076P2: RRID:AB_330924
Anti-Rabbit STAR 580	Abberior	Cat#ST580-1002
Anti-Mouse STAR 635	Abberior	Cat#ST635-1001
inVivoMAB anti-mouse PD-1	BioxCell	Clone RMPI 14;Cat# BE0146: RRID: AB_10949053
CD3 (IHC)	Abcam	Clone:NA: Cat# ab5690: RRID:AB_305055
CD4 (IHC)	Cell Signaling Technology	Clone D7D2Z;Cat# 25229: RRID:AB_2798898

CD8 (IHC)	Cell Signaling Technology	Clone D4W2Z; Cat# 98941: RRID:AB_2756376
Anti S1P antibody	Echelon Biosciences	Cat# Z-P300
Power Vision poly HRP anti rabbit	Leica Biosystems	Cat# PV6119: RRID:AB_1307590

Sup Table 5 List of Primers and siRNAs

Primer

Human PDL1	F 5′- CCA AGG CGC AGA TCA AAG AGA-3’<br>R 5′- AGG ACC CAG ACT AGC AGC A -3′
Mouse PDL1	F 5’-GACCAGCTTTTGAAGGGAAATG 3’<br>R 5’ CTGGTTGATTTTGCGGTATGG 3’
E2F1 chip primer	F 5’-ATT GGG CGT TTC TCT TGG T-3’<br>R 5’-GAG TAA GCG CCT GGG ATA TTT-3’
Human GUCY1A3	F 5’-TCAGCCCTACTTGTTGTACTCC-3’<br>R 5’-CAGAATAGCGATGTGGGAATCAC-3’
Human HLA-DMB	F 5’-ACCTGTCTGTTGGATGATGCT-3’<br>R 5’-CGCAAGGGGCCATCTTATTCT-3’
Human IL1A	F 5’-TGGTAGTAGCAACCAACGGGA-3’<br>R 5’-ACTTTGATTGAGGGCGTCATTC-3’

siRNA

TSG101	5’-GGU UAC CCG UUU AGA UCA A[dT][dT]-3’<br>5’-UUG AUC UAA ACG GGU AAC C[dT][dT]-3’
E2F1	SiRNA Id- SASI_Hs01_00162220 (SIGMA-AlDRICH)
ETS1	SiRNA Id- SASI_Hs01_00173246 (SIGMA-AlDRICH)
MYB	SiRNA Id- SASI_Hs01_00127047 (SIGMA-AlDRICH)
STAT1	SiRNA Id- SASI_Hs02_00343387 (SIGMA-AlDRICH)
IRF1	SiRNA Id- SASI_Hs01_00143090 (SIGMA-AlDRICH)
FOXH1	SiRNA Id- SASI_Hs01_00108856 (SIGMA-AlDRICH)
POU4F1	SiRNA Id- SASI_Hs02_00341153 (SIGMA-AlDRICH)
TFAP2A	SiRNA Id- SASI_Hs01_00080715 (SIGMA-AlDRICH)
HIF1A	SiRNA Id- SASI_Hs02_00332063 (SIGMA-AlDRICH)
CEBPA	SiRNA Id- SASI_Hs01_00058601 (SIGMA-AlDRICH)
SPHK1	ON-TARGET plus SMARTpool SiRNA-L-004172-00-0005 (DHARMACON)
S1PR1	ON-TARGET plus SMARTpool SiRNA-L-003655-00-0005 (DHARMACON)
S1PR2	ON-TARGET plus SMARTpool SiRNA-L-003952-00-0005 (DHARMACON)

MATERIALS AND METHODS

In vivo Extracellular vesicle treatment

RAB27a$^{-/-}$ ID8 cells and SPHK1$^{-/-}$ ID8 cells were orthotopically injected into the right ovary of C57BL/6 mice. EVs isolated by ultracentrifugation from culture media of ID8 cells, ID8 SPHK1$^{-/-}$ cells, and ID8 cells with SPHK1 overexpressed were injected into mice (20 μg/mouse) via the tail vein twice a week until the control mice became moribund.

Exogenous S1P treatment

S1P (Avanti Polar Lipids) was dissolved in fat-free bovine serum albumin (BSA) (4 mg/ml) in 1X phosphate buffer saline (PBS) (pH 7.4) at 125 mM concentration according to the protocol utilized by Panneer Selvam et al., 2015 [1]. The solution was added to culture medium at the required concentration.

Purification of EVs

Cells were cultured in serum-free media, and supernatants were collected at 48–72 h. EVs were purified from the supernatants by standard differential centrifugation [2]. In brief, the supernatants were centrifuged at 2,000g for 20 min to remove cell debris and dead cells; then they were centrifuged at 16,500g for 45 min. In turn, those supernatants were centrifuged at 100,000g for 2 h at 4 °C (Beckman Coulter, Optima XPN-100). The pelleted EVs were suspended in PBS and collected by ultracentrifugation at 100,000g for 2 h. Also, EVs were isolated from culture medium with Total Exosome Isolation Reagent (Invitrogen, Cat# 4478359).

To purify circulating EVs from blood using the exosome isolation kit, we first centrifuged cell-free plasma at 16,500g for 45 min (Eppendorf, 5418R) to pellet large membranous vesicles. EVs were then purified from the supernatant, using the Exosome Isolation Kit (Invitrogen, Cat# 4484450).

Measurement of S1P in plasma and SPHK1 activity

We used the Sphingosine 1-Phosphate ELISA Kit (Echelon, Salt Lake City, UT, USA) to quantify S1P in plasma samples from ovarian cancer patients and healthy controls, following the manufacturer's instructions. Sphingosine kinase activity was measured with the Sphingosine Kinase Activity Assay (Echelon, Salt Lake City, UT, USA) according to the manufacturer's instructions. In brief, protein extracts from EVs or WCL (5μg) were incubated in reaction buffer, 100 μm sphingosine, and 10μm ATP for 2h at RT, and then luminescence attached ATP detector was added to stop the kinase reaction. Kinase activity was measured using a luminometer (TECAN SPARK, Switzerland). All samples were prepared in triplicate, and the assay was repeated at least three times. Negative controls were prepared by heating samples at 90°C for 10 min to inactivate all enzymatic activity.

Measurement of S1P in culture media by Mass Spectrometry

S1P concentrations were quantified by the method of Berdyshev et. al. [3]. with 20 pg/μl of C_{17}S1P as the internal standard. After extraction and derivatization, bisacetylated S1P eluted from an Agilent Eclipse XDB-LB column (4.6 mm×150 mm, 5-μm particle) and detected using by an Agilent 6460 Triple Quadrupole mass spectrometer running in the negative ion mode and equipped with a JetStream source as previously reported [4]. Bisacetylated sphingolipids were eluted using the following gradients: 2-min hold of solvent A, ramp to 100% solvent B over 3 min, hold at 100% solvent B for 3 min, and then regenerate the column with solvent A for 4 min with the following settings: sheath gas temp =325°C, sheath gas flow =11 L/min, CE=13 V, fragmentor =210, acceleration voltage =7 V, capillary =3500 V, nozzle =1000 V. Solvent A = water/methanol/formic acid (20/80/0.5 v/v/v) mmol/L in ammonium formate. Solvent B = methanol/acetonitrile/formic acid (60/40/0.5 v/v/v) 5 mmol/L in ammonium formate. Multiple reaction monitoring transitions monitored were C_{17}S1P *m/z* 448/388 and S1P *m/z* 462/402 [5].

Western blotting

To prepare whole cell lysates, we washed cells twice with ice-cold PBS and lysed them on ice in 1x RIPA lysis buffer containing a freshly added protease inhibitor cocktail (Thermo Fisher Scientific Inc., Rockford, IL, USA) and 1 mM PMSF. To prepare tissue lysates, we homogenized tumor tissues in 1x RIPA lysis buffer over ice. After the homogenates were incubated for 30 min, lysates were collected by centrifugation at 4°C for 10 min at 10,000 rpm. The amount of total protein was determined using a BCA protein assay kit (Thermo Fisher Scientific Inc., Waltham, MA). An equal amount of total protein (30 µg) was resolved on precast 4%–12% SDS-PAGE gels (Biorad, Hercules, CA, USA), and the protein was transferred onto PVDF membranes and incubated with the desired primary antibodies. The preparation was then washed and incubated with HRP-conjugated secondary antibodies, and protein bands were detected with a chemiluminescence kit (Thermo Fisher Scientific Inc., Waltham, MA). List of the antibody used in this study is given is **S Table 4**.

siRNA transfection

Predesigned siRNAs for human SPHK1 (Cat# L-004172-00-0005), S1PR1 (Cat# L-003952-00-0005), and S1PR2 (Cat# L-003655-00-0005) were purchased from Dharmacon, Lafayette, CO. Negative siRNAs universal controls (siCont) were obtained from Sigma-Aldrich, which also supplied us with predesigned siRNAs. Transfections were performed using the Lipofectamine RNAiMAX transfection reagent (Thermo Fisher Scientific Inc., Waltham, MA). At 48 h post-transfection, cells were harvested for further analysis. siRNA sequences for all the genes are listed in **S Table 5**.

Tissue microarrays (TMAs) and immunohistochemistry (IHC)

SPHK1 and PD-L1 protein levels in human ovarian cancer tissues were analyzed using TMAs (Cat# OV1005b, US Biomax Inc., Rockville, MD). For this purpose, the slides were dewaxed in

xylene, and rehydrated through graded ethanol to distilled water. Antigen retrieval for the slide specimens was performed using IHC-Tek epitope retrieval solution and steamer set (IHC World, LLC.). The slides were then immersed in 3% H_2O_2 for 10 min to quench endogenous peroxidase, and then blocked with 10% goat serum for 1 h. The Vectastain ABC-AP Kit (Vector Labs, Burlingame, CA) and Vector Red Alkaline Phosphatase Substrate Kit I (Vector Labs, Burlingame, CA) were used for tissue staining as per the manufacturer's protocol. Both the SPHK1 primary antibody (Proteintech, Cat# 10670-I-AP) and the PD-L1 primary antibody (Proteintech, Cat# 17952-I-AP) were used at a 1:50 dilution. Following Vector red staining, the slides were counterstained with Harris modified hematoxylin (Thermo Fisher Scientific Inc., Rockford, IL), dehydrated with graded ethanol and xylene, and finally mounted with mounting medium. The TMA slides were then digitally scanned, using a Pannoramic 250 FLASH III scanner (3D HISTECH ltd. Version 2.0). Case Viewer software (3D HISTECH ltd. Version 2.0) was used to view and analyze the images. For other IHC, DAB sating was used according to the manufacturer's protocol (DAB Substrate Kit, Vectors Labs, Cat# SK-4100, Burlingame, CA).

To stain CD3, CD4, and CD8 in mouse tissue, we preheated slides at 60°C for 10 min and transferred them to a staining rack with xylene (3X 5 min each). Then the slides were hydrated through an alcohol gradient to distilled water. They were then placed in citrate buffer (Vector Labs) and then in antigen unmasking solution (Cat# H-3300) for 5 min. Next, the slides were placed in EDTA buffer (Sigma-Aldrich, Cat# E1161), and steamed for 45 min. When they had cooled, they were rinsed with PBST, and sections were incubated with blocking solution (Dako-dual endogenous enzyme block, Cat# S2003) for 5 min. After a rinse with PBST, the sections were incubated with primary antibodies CD3 (Abcam, Cat# ab5690, 1:500 dilution), CD4 (Cell Signaling, Cat# 25229, 1:100 dilution), and CD8 (Cell Signaling, Cat# 98941, 1:100 dilution) overnight. After a rinse with PBST, the slides were incubated with HRP-conjugated secondary antibody (Leica Biosystems, Power Vision poly HRP-anti rabbit, Cat# PV6119) for 1h, and then

DAB was applied. After a rinse with PBST, the slides were stained with hematoxylin, dehydrated with graded ethanol and xylene, and finally mounted with mounting medium (Sigma-Aldrich, Cat# 06522).

We performed S1P staining on frozen tissue sections according to Visentin B et. al. protocol [6]. Briefly, frozen tissue sections were place at RT for 20mins and fixed in 10% neutral buffered formalin for exactly 2 min. Slides were washed with water for 2 min. Endogenous peroxidase blocking was performed for 10 min in 0.3% (v/v) H_2O_2 in PBS. Slides were washed with PBS and blocked in 2x casein solution for 10min at RT. Then sections were incubated with S1P specific antibody (LT1002, Echelon Biosciences) overnight at 4˚C. ImmPRESS anti-mouse IgG polymer kit (Vector Laboratories, Cat# MP-7802) was used to develop the DAB staining.

Quantitative real time-PCR (qRT-PCR)

Total RNA was isolated from the cells using the RNeasy Mini Kit (Qiagen, Valencia, CA, USA), and first-strand cDNA was transcribed using iScript reverse transcription supermix (Biorad, Hercules, CA, USA). qRT-PCR was performed using CFX Connect Real-Time PCR systems (Biorad, Hercules, CA, USA) and SYBR Premix Ex Taq II (Biorad, Hercules, CA, USA). List of the qPCR primers used in the study is given as **S Table 5**.

A customized PCR array for human immune checkpoints was purchased from Qiagen (Valencia, CA, USA), and used according to the manufacturer's instructions. Data from the PCR array were analyzed using SABiosciences RT^2 Profiler PCR Data Analysis software (http://pcrdataanalysis.sabiosciences.com/pcr/arrayanalysis.php), and were considered significant at > 1.5 fold change and $p<0.05$. Five housekeeping genes, *B2M*, *HPRT1*, *RPLP0*, *GAPDH*, and *ACTB*, were used to normalize the data, and fold change was calculated relative to the control untreated OVACR5 cells.

Lentiviral SPHK1 knockdown

To create the lentiviral delivery system, HEK293T cells were transfected with packaging vectors pLP1, pLP2 and VSVG plasmids, including control empty vector pLKO.1 (Cat#SHC001V) and two different SPHK1-targeting short hairpin RNAs (shRNA) (TRC number 1: TRCN0000333028, Clone ID:NM_182965.2-1922s21c1; 2: TRCN0000333675, Clone ID: NM_182965.2-1104s21c1) purchased from Sigma-Aldrich (Saint Louis, MO). Competent lentiviruses were collected 48 h after transfection. HeyA8 cells were passaged to 40% confluence, and viral medium was added to them the next day along with 8 µg/ml of polybrene. The efficacy of individual SPHK1 shRNA constructs was checked by Western blot analysis. Puromycin (8 µg/ml; for 2 weeks) was used to select the most effective shRNA construct for generating a cell line in which SPHK1 was stably knocked down. The clones were picked and subjected to expansion culture for further selection. Western blot analysis was performed to identify the stable clone with the most efficiently downregulated SPHK1 protein. That clone was used in further experiments.

CRISPR/CAS9 knockdown of genes

SPHK1 and RAb27a genes knockout ID8 cells were engineered by Synthego (CA, USA) using CRISPR/CAS9 technology. The following guide sequence were used:

SPHK1- GCCGUGGGAGCAGUCCUCGA

RAB27a- CCUGCAGUUAUGGGACACGG

Transfections for overexpression

To establish the stable overexpression of SPHK1 and TSG101 in HeyA8 cells, we used Lipofectamine 2000 (Invitrogen, Carlsbad, CA) to transfect HeyA8 cells with a control vector or a pReceiver-M29 vector expressing SPHK1 (Cat# EX-H5064-M29), SPHK1 mutant 1 (PAAA) (Cat# CS-H5064-M29-01), SPHK1 mutant 2 (PTAA) (Cat# CS-H5064-M29-02), SPHK1 mutant 3 (ATAP) (Cat# CS-H5064-M29-03), and TSG101(Cat# EX-A6458-M29) were purchased from GeneCopoeia, Rockville, MD. 48 h after transfection, the cells were transferred to culture medium

containing G418 (800 μg/ml) for 2 weeks to ensure that the control sequences or SPHK1 would become stably incorporated. Western blotting was then performed to check GFP-SPHK1 and GFP-TSG101 expression. We also synthesized a viral vector to express mouse SPHK1 (pLCP-GFP+SPHK1-Neo), using a gene from the plasmid EX-Mm05392-M68 (GeneCopoeia, Rockville, MD). The viral plasmid was transfected into ID8 cells with polybrene (8 μg/μl) for 48 h, and selection was performed by culturing the cells in medium containing G418 (1000 μg/ml) for 1 week.

Proximity ligation assay

The DuoLink® In Situ Red Starter Kit Mouse/Rabbit (Sigma-Aldrich) was used to detect proximity between SPHK1 and TSG101 proteins according to the manufacturer's protocol. Briefly, HeyA8 cells were seeded in 8-well chamber slides (ibidi USA, Madison, WI, USA) and cultured overnight. The slides were then washed with cold 1×PBS, fixed in 4% paraformaldehyde for 30 min, and blocked with Duolink Blocking Solution in a pre-heated humidified chamber for 1 h at 4°C. The primary antibodies for detecting SPHK1 (Proteintech, Cat# 10670-I-AP) and TSG101 (Invitrogen Cat#, MA1-23296) were added, and the slides were incubated overnight at 4°C. The slides were then washed with 1×Wash Buffer A, and subsequently incubated with the PLA probes (1:5 dilution in antibody diluents) for 1h, then with Ligation-Ligase solution for 30 min, and finally with the Amplification-Polymerase solution for 100 min in a pre-heated humidified chamber at 37°C. Before imaging, the slides were washed with 1×Wash Buffer B and mounted with a coverslip, using Duolink In Situ Mounting Medium with DAPI. Fluorescence images were acquired with a confocal laser scanning microscope (LSM 510; Zeiss, Oberkochen, Germany).

STED imaging and image processing

Cells grown on cover slips (#1.5) were washed twice with PBS, and fixed with 2% paraformaldehyde (PFA) (15 min). The cells were rinsed with PBS three times, and permeabilized with 0.1% Triton X-100 (10 min). After washing, blocking was performed with 1X PBS/2% BSA

for 1 h. The primary antibodies of SPHK1 (Cat# 10670-I-AP, Proteintech, USA) and TSG101 (Cat# MA1-23296, Invitrogen, USA) were used for immunostaining in PBS for overnight at 4°C. After the slides were rinsed in PBS slides three times, cells were incubated with secondary antibodies goat anti-rabbit STAR 580 (Cat#ST580-1002, Abberior, Germany) and goat anti-mouse STAR 635 (Cat#ST635-1001, Abberior, Germany) for 1 h at RT. Cover slips were mounted using ProLong Gold Antifade Reagent (Life Technologies, Carlsbad, CA). Images were acquired with a 100X objective with 4.55 zoom, using a STED microscope (Leica). Image analysis was performed by Image J software, using the JACoP plugin.

Co-immunoprecipitation GFP Trap

HeyA8 cells stably expressing either GFP fusion proteins or SPHK1 variants were washed with PBS and then mechanically detached in PBS. The cells were pelleted by centrifugation at 500 × g for 3 min at 4 °C and rinsed twice with PBS. Cell lysis was achieved by adding lysis buffer (10 mM Hepes, 150 mM NaCl, 0.5 mM EDTA, 1% Triton X-100, 0.5% SDS, and proteinase inhibitor mixture, pH 7.5). We avoided Tris buffers because we wanted to use DTSSP cross-linker. Cleared lysates (17,000 × g, 15 min, 4°C) were incubated with GFP-nanobody agarose (GFP-Trap, Chromotek) for 1 h at 4°C. After the fusion proteins were bound to the beads, we applied 3 mM DTSSP cross-linker (Thermo Fisher Scientific) for 2 h at 4°C for nonspecific stabilization of protein–protein interactions. Finally, the beads were rinsed four times with Co-IP washing buffer (10 mM Hepes, 150 mM NaCl, 0.5 mM EDTA), and boiled in SDS-PAGE loading buffer for Western blot analysis.

Sucrose gradient for EVs purity

Culture medium was isolated and centrifuged at 4˚C (2000 rpm, 20 min) to remove cell debris. The supernatant was transferred into a new tube and centrifuged at 16,500rpm for 45 min at 4˚C to remove any large vesicles such as microvesicles. Supernatant was further transferred into

Beckman tubes (38.5 ml ultra-clear tubes) and ultracentrifuged at 4°C (100,000 g for 2 h) using a SW32 Ti rotor to pellet crude EVs, which were then resuspended in 100µl PBS.

We prepared stock solutions of sucrose ranging from 1%-60% in PBS and added them to a Beckman tube in the order of highest to lowest sucrose concentration. Crude EVs pellet was placed on top of the sucrose gradient and ultracentrifuged at 4°C (100,000g for 16 h) in a SW32 Ti rotor. We then collected 2 ml of each of the fractions, and ultracentrifuged them at 4°C (100,000g for 2 h). The isolated pellets were individually suspended in RIPA buffer, and western blotting was used to check the CD63, SPHK1, and ALIX expression.

Nanoparticle tracking analysis (NTA)

The EVs' size distribution and concentrations were determined by Nanoparticle Tracking Analysis (NTA), using the NanoSight LM10 instrument (Malvern Panalytical, Malvern, UK) with a 488 nm laser and NTA3.1 software. Three 30 s measurements were recorded for each sample, with automated analysis settings for blur, track length, and minimum expected particle size. The camera level was set at 12 and the detection threshold at 10.

Transcription factor array

OVACR5 cells were treated with 100 nM S1P for 6 h, and nuclear protein was extracted from treated and untreated cells. We measured the activity of 96 transcription factors in 15 µg of each protein extract, using the TF Activation Profiling Plate Array II (Signosis) according to manufacturer's instructions, Relevant TFs were selected by the fold change (>2 fold) between S1P-treated and untreated OVCAR5 cells.

Electron microscopy and immuno-gold staining of isolated exosomes

The exosome suspension was fixed for 30 min on ice by adding an equal volume of fixative (4% paraformaldehyde + 0.2% glutaraldehyde in 200 mM sodium phosphate buffer) to give a final concentration of 2% paraformaldehyde + 0.1% glutaraldehyde in 100mM buffer containing 3.5% sucrose and 0.5 mM $CaCl_2$. The EVs were then pelleted at 16,000 *g* for 10 min. The supernatant was discarded, and the pellet, still adhering to the Eppendorf centrifugation tube, was processed following the protocol of Bulreigh et. al. [7]. Essentially, the pellets were washed 3x20 min in 0.1M phosphate buffer containing 3.5% sucrose and 0.5 mM $CaCl_2$. Then unreacted aldehydes were quenched in 100 mM glycine in phosphate buffer for 1h on ice before the pellets were returned to PO_4 buffer. The pellets were then rinsed 4 times (for 15 min each time) in 100 mM tris maleate buffer + 3.5% sucrose pH 6.5 at 4°C and then in 2% uranyl acetate (w/v) in tris buffer pH6 for 2h at 4°C. After that, the pellets were given a final rinse 2x5 min in tris maleate buffer pH 6.5. They were then processed by the progressive lowering of temperature method of Berryman et al [8] into Lowicryl K4M resin, and polymerized by UV irradiation. Ultrathin sections (70 nm) were cut onto Formvar/carbon-coated grids. The grids were immunolabeled by floating them on 100mM phosphate buffer containing 5% BSA (PB-BSA) and then incubating them for 90 min with rabbit SPHK1 polyclonal antibody (Proteintech, Cat# 10670-I-AP) diluted 1:50. Non-immune rabbit polyclonal serum was used as the negative control. This step was followed by 3x5 min washes in PBS-BSA. The sections were then incubated with goat anti-rabbit IgG conjugated to 10nm colloidal gold for 90 min at room temp, rinsed in distilled water, stained with 2% aqueous uranyl acetate, and examined in a Hitachi H600 TEM at 75 kV.

T cell Isolation, treatment with EVs, and CFSE labeling, cytokine bead array

Human CD8+ T cells were purified from blood, using the EasySep Direct Human CD8+T Cell Isolation Kit (STEMCELL Technologies). The cells were stimulated with anti-CD3 (2µg/ml) and anti-CD28 (2 µg/ml) antibodies, and incubated with or without EVs for 72 h. The ratio of T cells and EVs was kept at 1:1500. The treated cells were then collected, stained, and analyzed by flow cytometry. Expression of Ki67, Granzyme B, PD-1, and FOXP3 were analyzed by flow cytometry.

To assay the proliferation of CD8+ T cells, we used CFSE dye. T cells were stained with 5μM CFSE and incubated at 37°C for 20 min. The reaction was stopped by adding 5 volumes of cold medium with10% FBS. The cells were then treated with EVs as above. Unstimulated CFSE-labeled cells served as a non-dividing control.

Stimulated T cells were either treated with EVs or S1P and cytokine bead array was performed according to manufacture protocol (BD, Cat# 560484, Human Th1/Th2/Th17 cytokine kit).

Luciferase reporter assay

Plasmid vector containing the promotor of PD-L1 was purchased from GeneCopoeia (Cat# HPRM40139-PG02 and transfected into cells by Lipofectamine-2000 reagent. The cells were treated with S1P or drugs at a given concentration for the next 24 h. Culture medium was then collected and centrifuged at 2000g for 10 min at 4°C, and 10 μl samples of supernatant were transferred into white-walled 96-well plates in triplicate. Luciferase intensity in each well was immediately measured, using a luminometer, as described in the Secrete-Pair Gaussia Luciferase Assay Kit (GeneCopoeia, Cat# LF061).

Preparation of ascites sample and flow cytometry

Ascites collected from euthanized mice were centrifuged at 2000 rpm. ACL lysis buffer was added according to the volume of the pellet (10 ml of buffer for 2 ml of pellet) and incubated for 5 min to lyse red blood cells. 1X PBS was added, and the preparation was centrifuged again to compact the cell pellet. The mixture was filtered through a 70 μm filter to collect tumor cells and TILs. These cells were then washed and counted, and $2x10^6$ cells were collected to be stained for flow cytometry. The single-cell suspension was first incubated with Fc block for 10 min, then live-dead staining was performed, and then the cells were washed with stain buffer (PBS+ 0.5% BSA). Cell-surface antigens were stained by co-incubation with antibodies for 30 min on ice followed by washing with stain buffer. Staining of intracellular FOXP3, Ki67, and Granzyme B was performed

with a BD Pharmingen kit (Cat# 562574) according to the manufacturer's protocol. Flow cytometry was performed on a Fortessa X20, and data were analyzed by FlowJo software. List of antibodies used for flow cytometry is given in S Table 4.

Activation of T cells with dendritic cells (DCs)

Femurs and tibias were isolated from wildtype C57BL/6 mice and flushed with 10% FBS containing RPMI medium. RBCs were lysed with ACK lysis buffer for 5 min; then medium was added, and cells were spun down. The cells were resuspended in 10% FBS containing RPMI at about 1–1.5x 10^6 per ml. Then FLT3L (100 ng/ml) was mixed into the suspension. Cells were plated into a 24-well plate at 1ml per well and cultured for the next 7 days. Meanwhile, ID8 cells were grown in a large flask and collected in PBS. 10x10^6 cells were suspended per 1.5 ml tube in PBS, and 5 cycles of freezing (dry Ice) and thawing (56°degree water bath) were performed. When 100% of the cells were dead (as confirmed with trypan blue staining), the tubes were spun at 1700g for 5 min and supernatants were collected and stored at −80°C for later use.

After 7 days of cell culture, the DC cells in each well were counted, and their percentage was checked using CD11c+MHCII+ antibodies to stain cells for flow cytometry. Around 60%–70% DCs were found per well. 180 µl of ID8 lysate (made as above) was added to each well, and the cells were maintained overnight in an incubator. The next day, LPS (100 ng/ml per well) was added to stimulate the DCs to mature. After about 6 h, DCs were collected and washed twice with PBS.

Activation of T cells with DC and IncuCyte experiment and flow cytometry

Spleen was isolated from one normal C57BL/6 mice and CD8 T cells were isolated using MojoSort™ Mouse CD8 T Cell Isolation Kit (Biolegend). T cells were mixed with DCs in the ratio of 5:1 (T cells:1 DCs) and divided in two group. 1) Untreated, 2) S1P (5 µM) and culture for next 3 days before performing IncuCyte experiment. Expression of Ki67, GranzymB and PD-1 was determined by flowcytometry on CD8+ T cells.

For IncuCyte experiment, ID8 cells (1000/well) were seeded in 96 well plate and T cells from both groups were added in ratio of 5:1 along with Annexin V IncuCyte reagent (red). This cocultures were then incubated in IncuCyte for three days and data was collected.

CHiP assay

We used the MatInspector (Genomatix) Analysis to determine if the PD-L1 promoter contains a binding site for the transcription factor E2F1. HeyA8 cells were treated with 100 nM S1P for 24 h. We performed the CHiP assay (using the protocol followed by Parashar D. et al [9] by adding E2F1 antibody (Cat No# 3742, CST) to both samples. Crude DNA (Input), control IGG, and no antibody samples were the controls. Primers amplifying the E2F1 binding region on the PD-L1 promoter were designed by PrimerQuest software (IDT). Real-time PCR were used to analyze the amplification of region when E2F1 binds to the PD-L1 promoter. Real-time PCR was used to amplify the binding region. Amplicons were also analyzed on agarose gel. The PCR results from the CHiP assay were analyzed by the fold enrichment method.

QUANTIFICATION AND STATISTICAL ANALYSIS

RNA-sequencing data from the TCGA and bioinformatics

The *R/Bioconductor* package *TCGAbiolinks* was used to retrieve RNA-Seq gene expression data (workflow HTSeq-Counts) of 376 patients in The Cancer Genome Atlas Ovarian Cancer (TCGA–OV) database (https://portal.gdc.cancer.gov/). Patients were ordered by the expression value of SPHK1, and the 3rd quartile value (863.8) was used as the cutoff to split patients into high and low groups. Thus, those with a raw count above 863.8 (i.e., the highest 25%) were in the high group, whereas those with a raw count below the cutoff were in the low group. Differential expression between the low and high SPHK1 groups was analyzed with the *edgeR-limma* suite of statistical methods. Genes with FDR <0.05 were considered to be differentially expressed (DE). Pre-ranked gene set enrichment analysis (GSEA) with DE genes pre-ranked by limma's t-statistics was performed to discover potential biological pathways associated with SPHK1 expression [10]. All analyses were performed using R software v4.0.4, and figures were produced

using the ggplot2 package (Wickham, 2016). A heatmap was drawn using the heatmap.3 function (https://github.com/obigriffith/biostar-tutorials/blob/master/Heatmaps/heatmap.3.R). The relative expression levels of selected genes between the high and low SPHK1 groups were analyzed with Student's *t*-test. $P < 0.05$ was considered statistically significant unless otherwise indicated.

We analyzed data from three biological replicates unless indicated otherwise in the figure legends. Statistical significance between two groups was defined as $*p<0.05$ $**p<0.01$ $***p<0.001$ $****p<0.0001$, as determined by unpaired Student's *t*-tests. We compared multiple groups by one-way analysis of variance (ANOVA) with statistical significance defined as $*p<0.05$ $**p<0.01$ $***p<0.001$ $****p<0.0001$ compared to control group as mentioned in the respective figure legends. Data are presented as mean ± standard error (SEM) as also indicated in the figure legends. To determine correlation coefficients, we calculated Mendor's correlation coefficients (r). GraphPad Prism 9 (GraphPad, San Diego, CA) was used to perform all statistical analyses and determine *p*-values.

References

[1] S. Panneer Selvam, R. M. De Palma, J. J. Oaks, N. Oleinik, Y. K. Peterson, R. V. Stahelin, E. Skordalakes, S. Ponnusamy, E. Garrett-Mayer, C. D. Smith, B. Ogretmen, *Sci Signal* **2015**, *8* (381), ra58, https://doi.org/10.1126/scisignal.aaa4998.

[2] G. Chen, A. C. Huang, W. Zhang, G. Zhang, M. Wu, W. Xu, Z. Yu, J. Yang, B. Wang, H. Sun, H. Xia, Q. Man, W. Zhong, L. F. Antelo, B. Wu, X. Xiong, X. Liu, L. Guan, T. Li, S. Liu, R. Yang, Y. Lu, L. Dong, S. McGettigan, R. Somasundaram, R. Radhakrishnan, G. Mills, Y. Lu, J. Kim, Y. H. Chen, H. Dong, Y. Zhao, G. C. Karakousis, T. C. Mitchell, L. M. Schuchter, M. Herlyn, E. J. Wherry, X. Xu, W. Guo, *Nature* **2018**, *560* (7718), 382, https://doi.org/10.1038/s41586-018-0392-8.

[3] E. V. Berdyshev, I. A. Gorshkova, J. G. Garcia, V. Natarajan, W. C. Hubbard, *Anal Biochem* **2005**, *339* (1), 129, https://doi.org/10.1016/j.ab.2004.12.006.

[4] A. Ossoli, S. Simonelli, M. Varrenti, N. Morici, F. Oliva, M. Stucchi, M. Gomaraschi, A. Strazzella, L. Arnaboldi, M. J. Thomas, M. G. Sorci-Thomas, A. Corsini, F. Veglia, G. Franceschini, S. K. Karathanasis, L. Calabresi, *Arterioscler Thromb Vasc Biol* **2019**, *39* (5), 915, https://doi.org/10.1161/ATVBAHA.118.311987.

[5] a) M. J. Taylor, R. Menzies, L. J. MacMillan, H. E. Whyte, *Electroencephalogr Clin Neurophysiol* **1987**, *68* (1), 20, https://doi.org/10.1016/0168-5597(87)90066-9; b) M. H. Lee, K. M. Appleton, H. M. El-Shewy, M. G. Sorci-Thomas, M. J. Thomas, M. F. Lopes-Virella, L. M. Luttrell, S. M. Hammad, R. L. Klein, *J Lipid Res* **2017**, *58* (2), 325, https://doi.org/10.1194/jlr.M070706.

[6] B. Visentin, G. Reynolds, R. Sabbadini, *Methods Mol Biol* **2012**, *874*, 55, https://doi.org/10.1007/978-1-61779-800-9_5.

[7] B. A. Burleigh, C. W. Wells, M. W. Clarke, P. R. Gardiner, *J Cell Biol* **1993**, *120* (2), 339, https://doi.org/10.1083/jcb.120.2.339.

[8] M. A. Berryman, R. D. Rodewald, *J Histochem Cytochem* **1990**, *38* (2), 159, https://doi.org/10.1177/38.2.1688894.

[9] D. Parashar, A. Geethadevi, M. R. Aure, J. Mishra, J. George, C. Chen, M. K. Mishra, A. Tahiri, W. Zhao, B. Nair, Y. Lu, L. S. Mangala, C. Rodriguez-Aguayo, G. Lopez-Berestein, A. K. S. Camara, M. Liang, J. S. Rader, R. Ramchandran, M. You, A. K. Sood, V. N. Kristensen, G. B. Mills, S. Pradeep, P. Chaluvally-Raghavan, *Cell Rep* **2019**, *29* (13), 4389, https://doi.org/10.1016/j.celrep.2019.11.085.

[10] a) A. Subramanian, P. Tamayo, V. K. Mootha, S. Mukherjee, B. L. Ebert, M. A. Gillette, A. Paulovich, S. L. Pomeroy, T. R. Golub, E. S. Lander, J. P. Mesirov, *Proc Natl Acad Sci U S A* **2005**, *102* (43), 15545, https://doi.org/10.1073/pnas.0506580102; b) V. K. Mootha, C. M. Lindgren, K. F. Eriksson, A. Subramanian, S. Sihag, J. Lehar, P. Puigserver, E. Carlsson, M. Ridderstrale, E. Laurila, N. Houstis, M. J. Daly, N. Patterson, J. P. Mesirov, T. R. Golub, P. Tamayo, B. Spiegelman, E. S. Lander, J. N. Hirschhorn, D. Altshuler, L. C. Groop, *Nat Genet* **2003**, *34* (3), 267, https://doi.org/10.1038/ng1180.
